# Signs of innate immune activation and premature immunosenescence in psoriasis patients

**DOI:** 10.1038/s41598-017-07975-2

**Published:** 2017-08-08

**Authors:** Liisi Šahmatova, Elena Sügis, Marina Šunina, Helen Hermann, Ele Prans, Maire Pihlap, Kristi Abram, Ana Rebane, Hedi Peterson, Pärt Peterson, Külli Kingo, Kai Kisand

**Affiliations:** 1 Department of Dermatology, University of Tartu, Tartu, Estonia; 2 Dermatology Clinic, Tartu University Hospital, Tartu, Estonia; 3 Institute of Computer Science, University of Tartu, Tartu, Estonia; 4 Quretec Ltd, Tartu, Estonia; 5 Institute of Biomedicine and Translational Medicine, University of Tartu, Tartu, Estonia

## Abstract

Psoriasis is a chronic inflammatory disease that affects skin and is associated with systemic inflammation and many serious comorbidities ranging from metabolic syndrome to cancer. Important discoveries about psoriasis pathogenesis have enabled the development of effective biological treatments blocking the T helper 17 pathway. However, it has not been settled whether psoriasis is a T cell-mediated autoimmune disease or an autoinflammatory disorder that is driven by exaggerated innate immune signalling. Our comparative gene expression and hierarchical cluster analysis reveal important gene circuits involving innate receptors. Innate immune activation is indicated by increased absent in melanoma 2 (*AIM2*) inflammasome gene expression and active caspase 1 staining in psoriatic lesional skin. Increased eomesodermin (*EOMES*) expression in lesional and non-lesional skin is suggestive of innate-like virtual memory CD8+ T cell infiltration. We found that signs of systemic inflammation were present in most of the patients, correlated with the severity of the disease, and pointed to IL-6 involvement in the pathogenesis of psoriatic arthritis. Among the circulating T cell subpopulations, we identified a higher proportion of terminally differentiated or senescent CD8+ T cells, especially in patients with long disease duration, suggesting premature immunosenescence and its possible implications for psoriasis co-morbidities.

## Introduction

Plaque psoriasis is a common chronic complex disease^1^ with a prevalence of approximately 0.91–8.5% in adults^2^. Although it affects the skin, psoriasis is not a skin-restricted disease but a systemic inflammatory disorder^1, 3, 4^. Furthermore, many co-morbidities are associated with psoriasis, e.g., obesity, diabetes, cardiovascular diseases, non-alcoholic fatty liver disease, Crohn’s disease, lymphoma, cancer, anxiety and depression, many of which can be the result of persistent inflammation in the body^1, 3^. The phenotypic features of plaque psoriasis are variable: the severity of the disease, the involvement of joints or nails or the onset age of the disease may be related to different pathogenic mechanisms. Nevertheless, it has been proven that inflammation in the skin is governed by cytokines IL-17, IL-22, IL-23, IFN-γ and tumour necrosis factor (TNF)-α, which synergistically induce keratinocyte hyperproliferation, secretion of chemokines and antimicrobial peptides (AMP)^1, 5, 6^. Changes are most prominent in the lesional skin of psoriasis, but there are also clear signs of inflammation and immune cell infiltration in seemingly healthy skin of the patients^7–9^. Research on psoriasis has enabled the development of effective drugs that block the T helper (Th)17 cytokine-related pathway that is now established as central in the pathogenesis of this disease^1, 6, 10–12^.

However, it is still debated whether psoriasis is autoimmune, e.g., caused by autoantigen-specific T cells, or rather autoinflammatory due to excessive stimulation of innate immune receptors. The aim of our study was to find support for either hypothesis using marker gene expression analysis in skin biopsy samples and active caspase 1 staining in skin. Moreover, as the long-term consequences of systemic inflammation on circulating immune cells are not known in psoriasis patients, we immunophenotyped T cell subpopulations in patients with different disease durations.

Our results suggest that innate immune receptors and inflammasomes as well as innate immune cells are important in psoriasis pathogenesis and that systemic inflammation throughout the course of the disease may lead to premature immunosenescence of the circulating CD8+ T cell compartment.

## Results

### Inflammatory cytokines, chemokines and AMPs are upregulated, but *CCL27* is downregulated in psoriatic lesions

**Figure 1:**
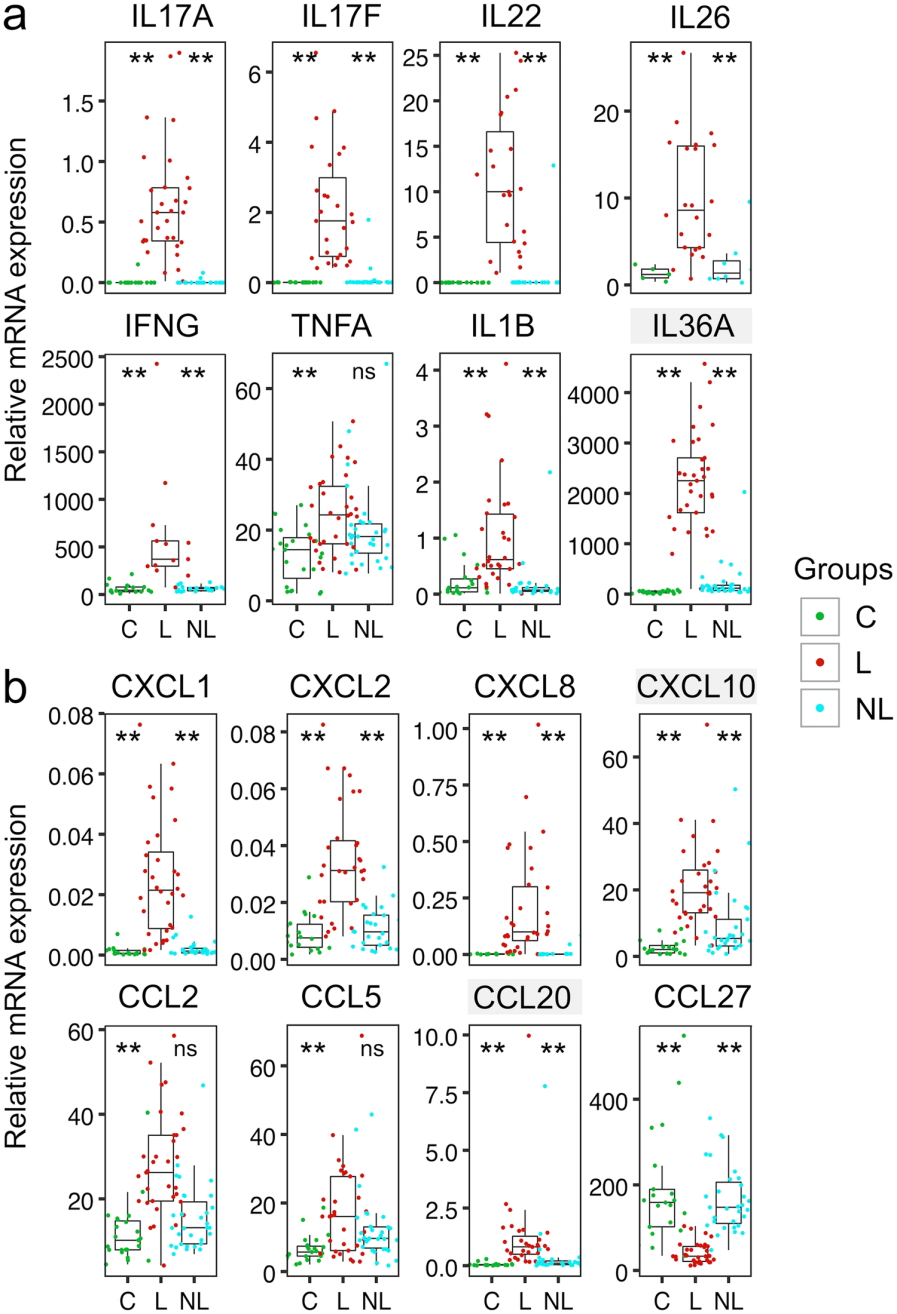
Inflammatory cytokine and chemokine expression is elevated in psoriatic lesions. QRT-PCR was used to quantify the expression of inflammatory cytokines (**a**) and chemokines (**b**) relative to *ACTB* in skin biopsy samples obtained from lesional (L) and non-lesional (NL) skin of psoriasis patients as well as from age-matched control individuals (C). The box-and-whisker plots depict median (central line), interquartile range (IQR, Q1-Q3, box), Q3 + 1.5 × IQR (upper whisker) and Q1 − 1.5 × IQR (lower whisker). Stars above the groups C and NL depict their significance level from L samples. **P < 0.001, *P < 0.05. Grey shading behind the gene name indicates statistically significant differences between NL and C biopsy samples (for IL36A *P* = 1.70*10^−5^, CXCL10 *P* = 0.0019, CCL20 *P* = 0.022).

The summary of gene expression analysis from skin biopsy samples derived from psoriatic lesions, non-lesional skin and control individuals can be found in Supplementary Fig. S1 and Supplementary Table S1. We confirmed that Th17 cytokine gene expression (*IL17A*, *IL17F*, *IL22*, *IL26*) was significantly increased in psoriatic lesional skin in comparison to non-lesional and control skin (Fig. 1a). mRNA from other proinflammatory cytokines associated with psoriasis pathogenesis, *IFNG*, *TNFA* and IL-1 family members *IL1B* and *IL36A* were also markedly elevated in psoriatic lesional skin (Fig. 1a). In addition, we looked for the expression of several cytokine receptor genes (Supplementary Fig. S2), of which only *IL22RA1* showed elevated mRNA signals in psoriatic skin. IL-17A is known to induce the production of the neutrophil-attracting chemokines *CXCL1*, *CXCL2* and *CXCL8* from keratinocytes^13^. Indeed, their expression was significantly elevated in the lesional skin of psoriasis patients (Fig. 1b). The expression of other proinflammatory chemokines (*CCL2*, *CCL5* and *CCL20*) recruiting various inflammatory cells, including monocytes, dendritic cells and Th17 cells, was also increased (Fig. 1b). In line with previous studies, *CCL27* that attracts CCR10+ cells^9, 14, 15^ was significantly downregulated in psoriatic skin (Fig. 1b). *CXCL10* is upregulated by IFNs – its overexpression in psoriatic lesions underlines the importance of IFNs in disease pathogenesis (Fig. 1b). Th17 cytokines are potent inducers of AMP expression: *S100A8*, *S100A9*, *PI3* and *LCN2* were significantly upregulated in psoriatic skin (Supplementary Fig. S3). These results are in line with multiple previous studies confirming the central role of Th17 cytokines in psoriasis^1^.

### *EOMES* expression is elevated in psoriatic skin

**Figure 2:**
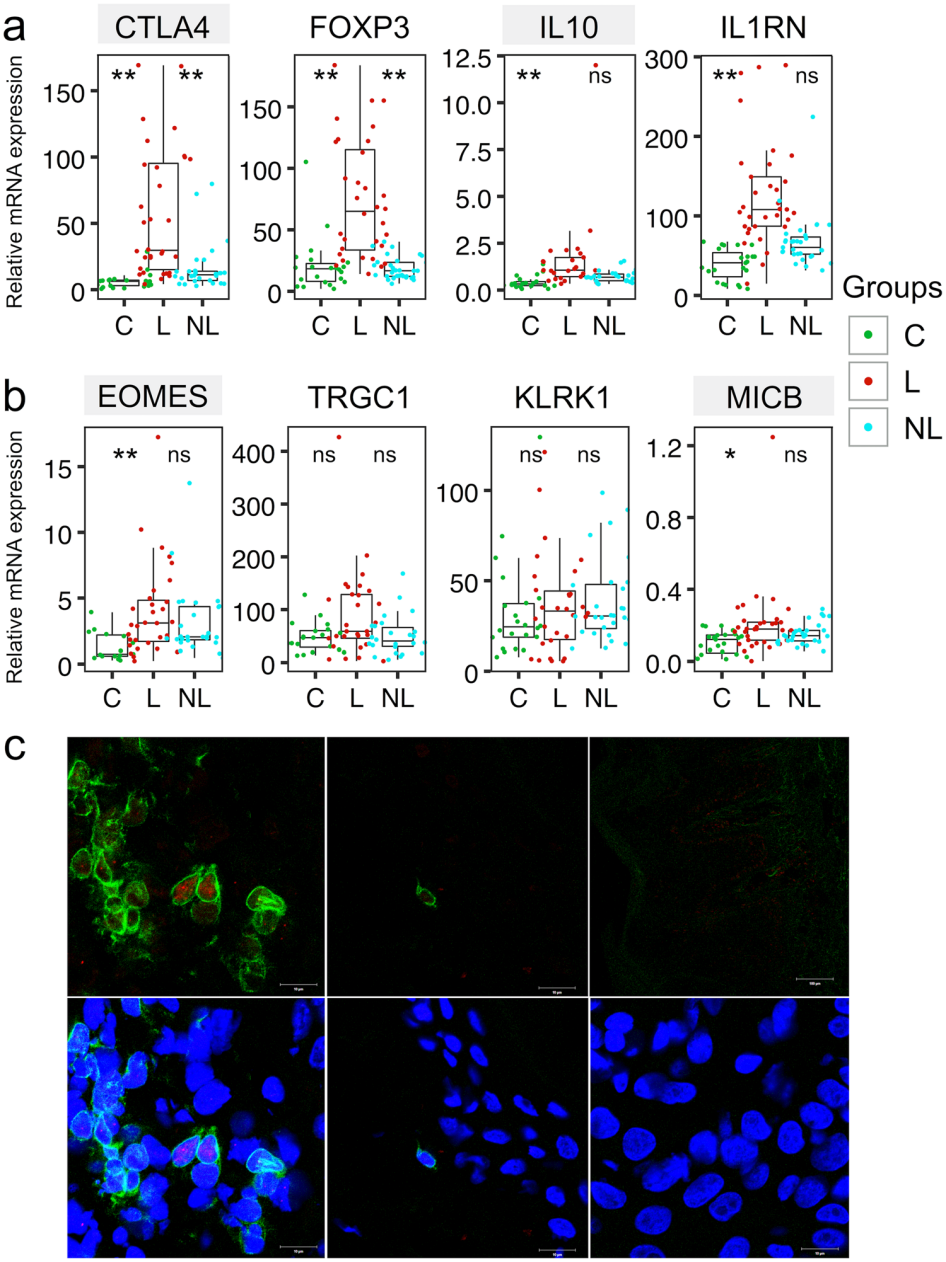
Several immunoregulatory genes and marker transcription factors are upregulated in psoriatic skin. mRNA expression of several immunoregulatory genes (**a**) and genes specific for different cytotoxic effector cells (**b**) relative to *ACTB* in skin biopsy samples obtained from lesional (L) and non-lesional (NL) skin of psoriasis patients and from age-matched control individuals (C). The description of the box-and-whiskers and stars can be found in the legend of Fig. 1. Gray shading behind the gene name indicates statistically significant differences between NL and C samples (*P* < 0.001). (**c**) Fluorescence microscopic images illustrate CD3 expression (green) and Eomesodermin staining (red) in psoriatic lesional skin biopsy frozen sections (left). The middle panels represent healthy control skin and right-hand panels negative control staining (secondary antibodies only). The lower panels include 4′,6-diamidine-2′-phenylindole dihydrochloride (DAPI) for counterstaining cell nuclei. White bar represents 10 µm.

Inflammation is counterbalanced by negative regulators of immune responses. Indeed, the expression of *FOXP3*, coinhibitory molecule *CTLA4*, *IL10* and *IL1RN* were markedly elevated in psoriatic lesions (Fig. 2a). However, FOXP3+ regulatory T cells (Treg) can lose their regulatory capacity in the inflammatory environment – they reportedly make IL-17A in psoriatic skin^16^.

There are multiple effector cells that could directly mediate the damage of keratinocytes, such as cytotoxic T cells, NK and γδ T cells. While we did not see any statistically significant difference in NK cell-specific gene *KLRK1* expression or TCR gamma chain (*TRGC1*) expression, we noticed significantly higher levels of the transcription factor *EOMES* mRNA in psoriatic lesions (Fig. 2b). *EOMES* is expressed in NK cells, effector cytotoxic T cells and virtual memory “innate-like” CD8+ T cells^17–19^. Immunofluorescent microscopy of psoriatic skin sections confirmed that a fraction of CD3 + T cells indeed contained Eomesodermin in their nuclei (Fig. 2c, left) while control skin sections (Fig. 2c, middle) contained very few T cells. Interestingly, we found a moderate increase in stress molecule *MICB* expression in psoriatic skin that makes the cells vulnerable to NK cell attack^20, 21^ (Fig. 2b).

### Innate receptors and inflammasomes in psoriatic lesions

**Figure 3:**
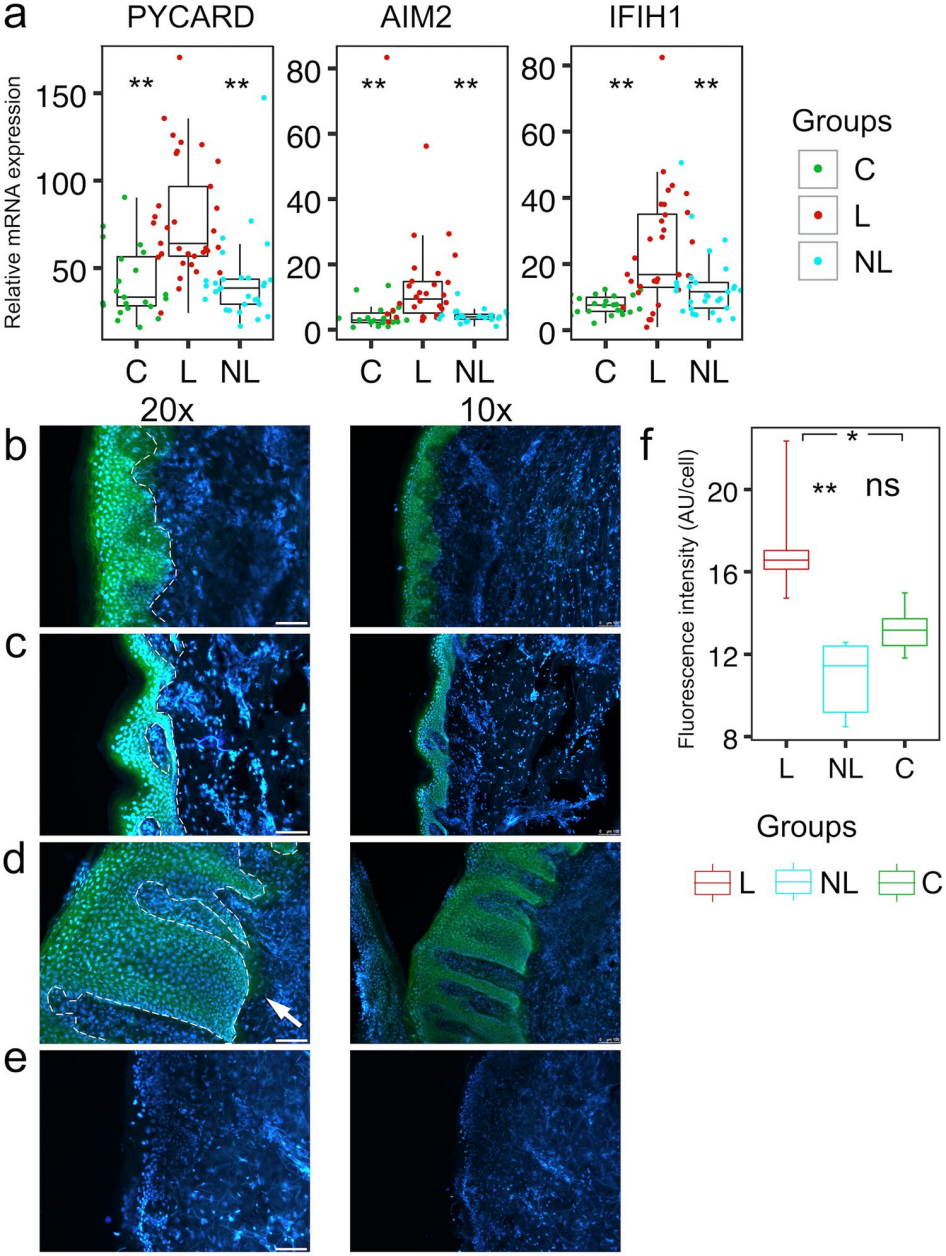
Innate receptors’ upregulation and inflammasome activation in psoriatic lesions. (**a**) mRNA expression of genes encoding innate receptors relative to *ACTB* in skin biopsy samples obtained from lesional (L) and non-lesional (NL) skin of psoriasis patients and from age-matched control individuals (C). The description of the box-and-whiskers and stars can be found in the legend of Fig. 1. (**b–e**) Fluorescence microscopic images illustrate caspase-1 activation as detected with FAM-FLICA Assay Kit (green) in control skin (**b**) psoriatic non-lesional skin (**c**) or psoriatic lesional skin (**d**) biopsy frozen sections with DAPI for counterstaining cell nuclei. (**e**) Represent control slides. White bars in 20x images represent 75 µm. White dotted lines indicate the basal membrane. (**f**) Green fluorescence intensity was measured from the slides in fixed areas of stratum spinosum (from slides of two different individuals and 2–3 different areas per slide) and divided by the number of cells per the area.

Next, we studied components of the autophagosome (*WIPI1* ^22^) and several innate receptors *(IFIH1, AIM2*) and inflammasome components (*NLRP1, NLRP3, PYCARD, CASP1*) in the skin of psoriasis patients in comparison with control subjects, as autoinflammatory diseases are often associated with inflammasome activation in response to stress signals (Fig. 3a, Supplementary Fig. S4). Moreover, *IL1B*, that showed higher expression in psoriatic lesions, relies on inflammasome activation to acquire its biologically active form. *WIPI1*, *NLRP1*, *NLRP3* and *CASP1* expression levels were not significantly different in the studied groups (Supplementary Fig. S4). However, the *IFIH1*, *AIM2* and *PYCARD* genes encoding inflammasome adaptor protein ASC showed increased expression in psoriatic skin (Fig. 3a). To determine whether the elevated expression of inflammasome components could be associated with increased inflammasome activation and active caspase 1 accumulation in psoriatic lesions, we used the fluorochrome-labelled inhibitor of caspases (FLICA) reagent, which forms a covalent bond with active caspase 1. FLICA staining of healthy skin cryosections localized exclusively to the epidermis with highest signals in granular layer (Fig. 3b), indicating caspase 1 activation during normal epidermal maturation. While psoriatic non-lesional skin (Fig. 3c) did not substantially differ from healthy control skin, FLICA staining was detectable throughout the thickened psoriatic epidermis in the lesional skin and covered some dermal areas close to the epidermis (white arrow in Fig. 3d). Due to intercellular edema in psoriatic epidermis the number of cells per fixed section area was lower, and therefore the green fluorescence intensity per keratinocyte was significantly higher in psoriatic lesion in comparison to healthy skin or nonlesional skin of psoriasis patients (Fig. 3f). These results strongly suggest the increased activation of innate immunity in psoriatic lesions.

**Figure 4:**
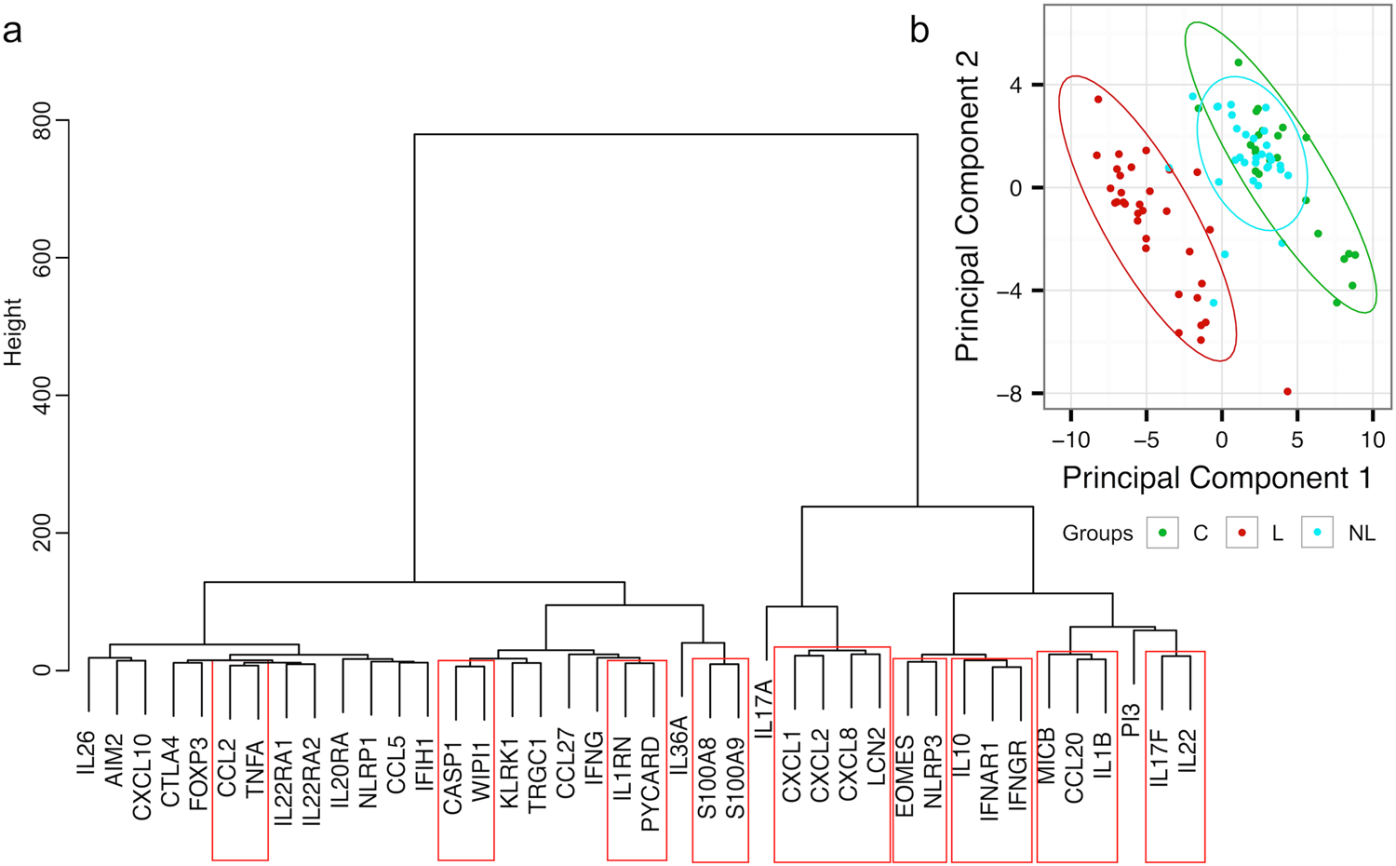
Hierarchical clustering and PCA of gene expression data. (**a**) Euclidian distance and ward linkage were used as parameters to cluster gene expression profiles. Strong clusters (depicted with boxes around gene names) were selected with pvclust. (**b**) PCA of gene expression data from biopsy samples derived from lesional (L) and non-lesional (NL) skin of psoriasis patients and from age-matched control individuals (C).

Next, we performed a hierarchical cluster analysis to find genes with a similar expression pattern. By using a cluster dendrogram, nine gene clusters were statistically significantly separated (Fig. 4a).

### Gene expression in psoriatic non-lesional skin reveals immune cell infiltration

Principal component analysis (PCA) of the gene expression data readily segregated lesional skin samples from the other studied groups by the first two components (Fig. 4b). Psoriatic non-lesional skin mostly overlapped with control skin except for three non-lesional samples that appeared in or close to the lesional skin cluster (Fig. 4b). Two of these belonged to patients with very high psoriasis area and severity index (PASI) scores (44 and 28). Although PCs did not distinguish healthy skin from psoriatic non-lesional skin, we were able to detect significant gene expression alterations in seemingly healthy skin in psoriasis patients (statistically significant differences between control and non-lesional samples are indicated with grey shading of the gene labels in Figs 1, 2 and S2). While the marker cytokines of psoriasis were virtually non-detectable in non-lesional skin, chemokines *CXCL10* and *CCL20*, the respective chemoattractants for Th1 and Th17 cells, were significantly increased. Indeed, some effector cell infiltration to non-lesional skin can be indicated by higher *EOMES* expression (Fig. 2b). Importantly, the immune-regulatory molecules prevail in non-lesional skin over the Th17 cytokines that remained mostly under the detection limit: *CTLA4* and *IL10* showed significantly elevated expression in non-lesional samples compared to control skin (Fig. 2a).

### Gene expression signatures of psoriasis subtypes

Next, we aimed to find gene expression signatures for different phenotypic features of psoriasis (Supplementary Fig. S5). Interestingly, gene expression in the psoriatic lesions was rather uniform in patients with different subtypes of psoriasis. Only *TNFA* expression was significantly lower in patients with complicating psoriatic arthritis. In contrast, gene expression in non-lesional skin was more variable in psoriasis subgroups. Moderate to severe psoriasis (PASI ≥ 12) was associated with higher *S100A9* and *CXCL1* expression levels in seemingly healthy skin than in patients with mild psoriasis (PASI < 12). Patients with nail involvement had higher *CXCL10* expression; longer duration of the disease was associated with further decrease of *CCL27* in areas that were not affected with psoriatic rash; and psoriatic arthritis was associated with lower *IFIH1* expression. To conclude, not only lesional skin but also the non-lesional skin of psoriasis patients reveals signs of inflammation that are associated with the variable clinical features of psoriasis.

### Plasma level of cytokines is consistent with systemic inflammation

**Figure 5:**
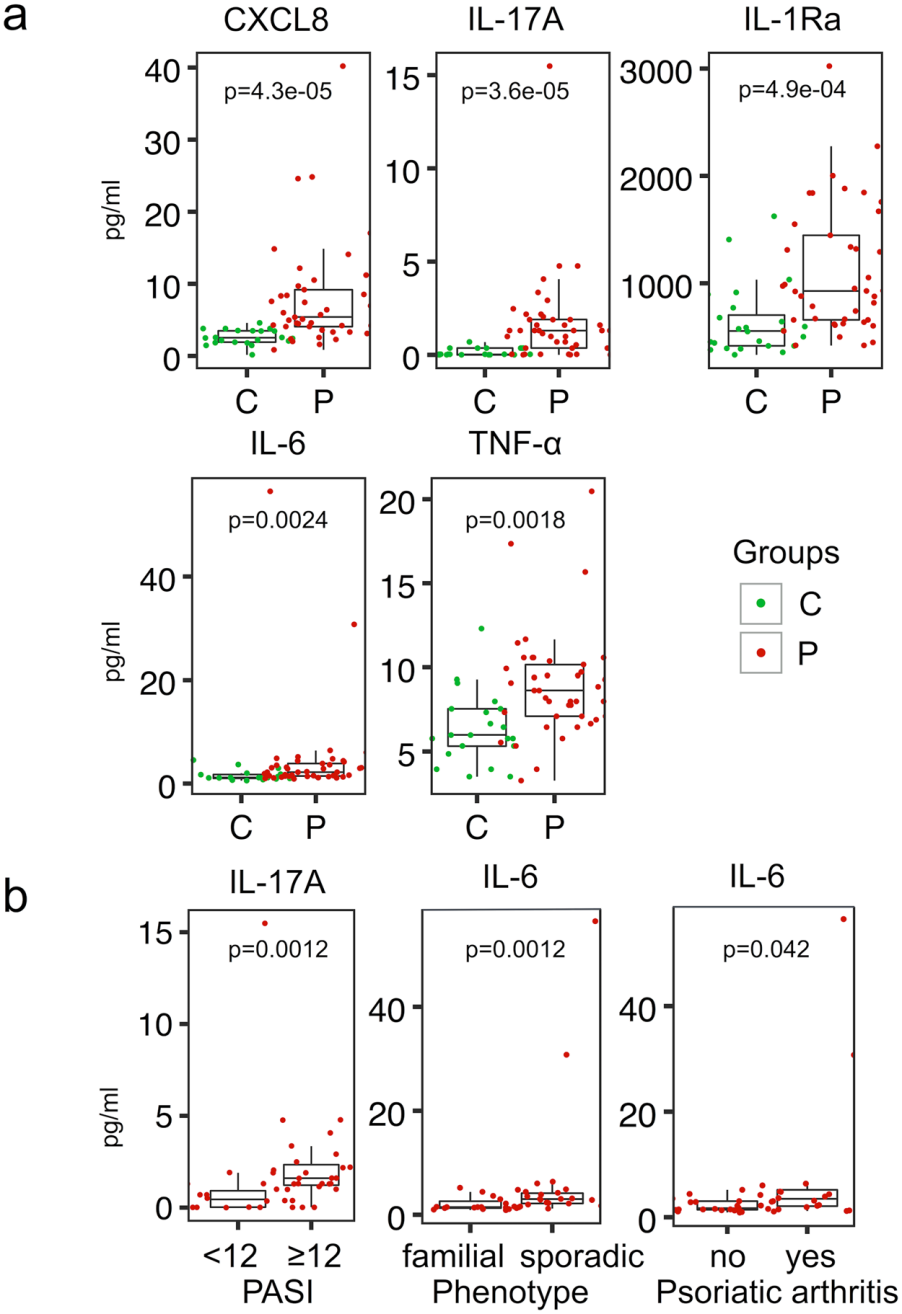
Comparison of circulating immune mediators in psoriasis patients and control individuals. (**a**) The Luminex method was used to determine the cytokine and chemokine concentrations in plasma samples of psoriasis patients (P) and control individuals (C). (**b**) Comparison of circulating cytokine levels in psoriasis patients with different clinical features. The box-and-whisker plots depict median (central line), interquartile range (IQR, Q1-Q3, box), Q3 + 1.5 × IQR (upper whisker) and Q1 − 1.5 × IQR (lower whisker).

To further study the signs of systemic inflammation in psoriasis, we measured the concentration of several cytokines and chemokines in plasma samples of all participants using the Luminex method. The levels of IL-17A, IL-6, TNF-α, IL-1Ra and CXCL8 were significantly higher in the plasma of psoriatic patients in comparison to control subjects (Fig. 5a). The IL-17A concentration was higher in moderate to severe psoriasis, while higher IL-6 levels were associated with joint involvement and sporadic form of psoriasis (Fig. 5b). Taken together, signs of systemic inflammation are present in most of the patients, correlate with the severity of the disease, and point to IL-6 involvement in the pathogenesis of psoriatic arthritis.

### CD8+ T cell compartment reveals signs of premature senescence in psoriasis

**Figure 6:**
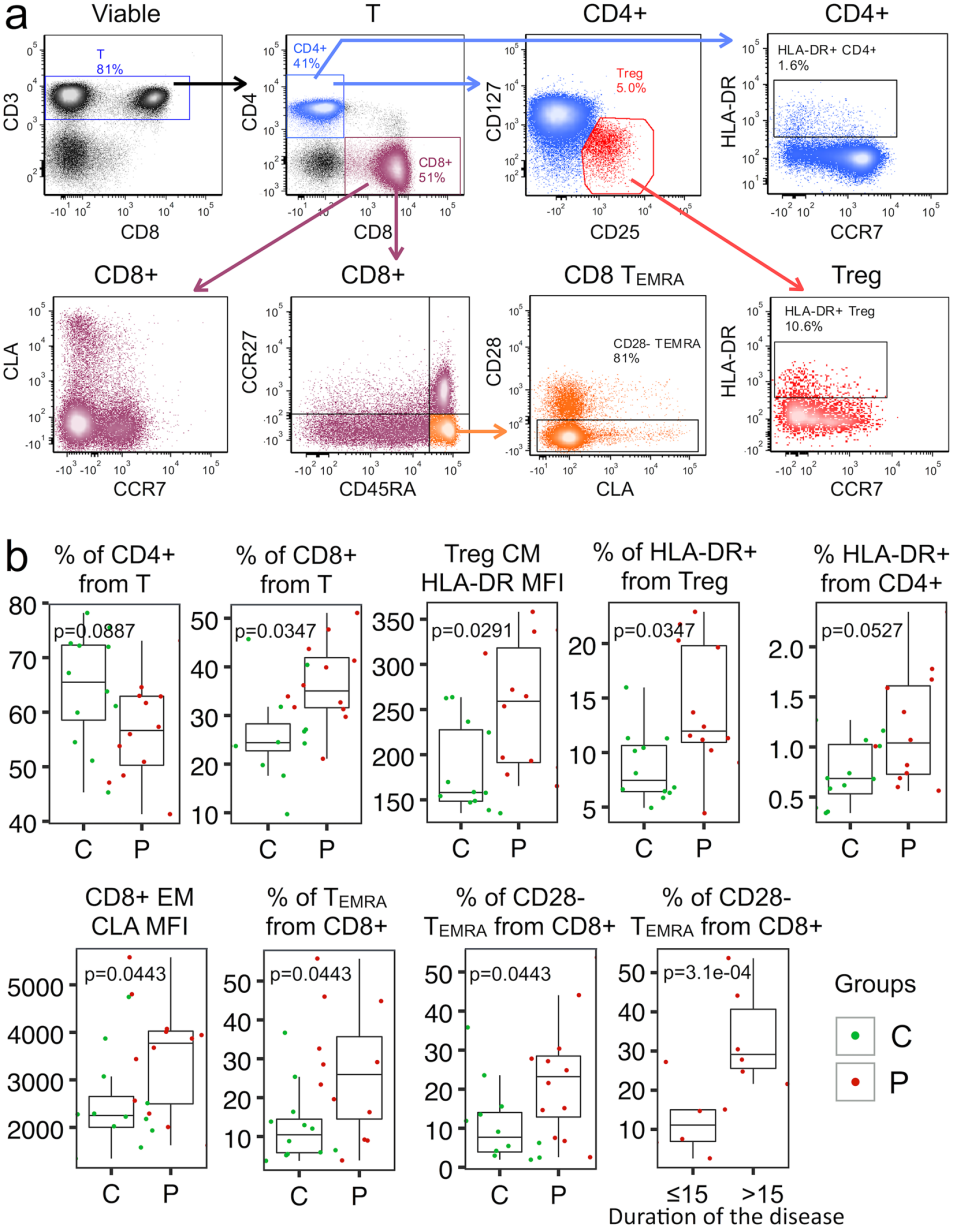
Characteristics of circulating T cell subpopulations in psoriasis patients (P) and control individuals (C). (**a**) Representative FACS plots are shown. More detailed gating strategy can be found in Supplementary Material. (**b**) The box-and-whisker plots depict median (central line), interquartile range (IQR, Q1-Q3, box), Q3 + 1.5 × IQR (upper whisker) and Q1 − 1.5 × IQR (lower whisker). CM – central memory (CCR7+, CD45RA−), Treg – regulatory T cell (CD4+, CD25+, CD127low) EM – effector memory (CCR7−, CD45RA−), CLA – cutaneous lymphocyte antigen, MFI – median fluorescence intensity, T_EMRA_ – effector memory T cell with CD45RA expression.

Sustained inflammation is supposed to have detrimental effects on the immune cells, as seen during inflammageing^23^. To study changes in the T cell compartment, we performed flow-cytometric immunophenotyping of various T cell subpopulations (Supplementary Table S2) in a subgroup of 12 patients with psoriasis (mean age 39 years) and age- and gender-matched control individuals (n = 12, mean age 40 years). The gating strategy is depicted in Supplementary Fig. S6. Among Tregs, we noted higher proportions of HLA-DR+ cells indicating a higher activation status of these cells (Fig. 6), and a similar tendency was seen for CD4+ T cells (significance was lost after multiple comparison correction). The proportion of CD8+ T cells was increased in psoriasis patients, and among the effector memory (EM) subpopulation, cutaneous lymphocyte antigen (CLA) expression was increased. Among CD8+ cells, the terminally differentiated or senescent T cells (T_EMRA_ as well as CD28- T_EMRA_) had higher proportions in psoriasis patients. Moreover, the senescent population of the T cells was more pronounced in patients with a longer duration of the disease (≥15 years). To conclude, the T cells of psoriasis patients reveal signs of excessive immune activation as well as features of premature immunosenescence.

## Discussion

Psoriasis is a prototypical Th17 cytokine-mediated chronic inflammatory disease^11, 24^, and biologicals blocking the Th17 pathway have shown high efficacy in psoriasis treatment^6, 7, 10, 25^. Nevertheless, the data about the primary cellular source of Th17 cytokines have remained controversial. While Th17 cells were the first suspects for making IL-17A in psoriatic lesional skin, several recent studies have pointed to CD8+ and γδ T cells as an important IL-17A source in this disease^26–30^. We were unable to see any significant difference in *TRGC1* expression in psoriatic skin that does not rule out an increase in the proportion of IL-17A secreting cells. Indeed, Harden *et al*.^31^ found several γ-chain sequences that were shared by psoriasis patients’ skin samples. In addition to γδ T cells, neutrophils and mast cells have been shown to release IL-17A during extracellular trap formation (NETosis) in psoriasis and to be the major IL-17A+ cells in psoriatic skin^32^. IL-22 and IL-17F cellular sources in psoriasis have been less studied and possibly assumed to be closely co-regulated with IL-17A. According to our data, *IL17F* and *IL22*, which together formed a significant cluster (Fig. 4a), were relatively far from *IL17A* in the dendrogram, meaning that these interleukins may rely more on other cell sources (e.g., Th17 and Th22 cells) than on neutrophils for their production. *IL17A* was the closest to the cluster that contained the neutrophil-attracting chemokines *CXCL1*, *CXCL2* and *CXCL8* as well as the antimicrobial *LCN2*, which is perfectly in line with the well-known role of IL-17A in their induction^1, 6, 33^. However, *PI3*, another AMP, as well as *CCL20*, which is needed for the recruitment of CCR6+ Th17 cells, seem to be co-induced by *IL22*, *IL17F* and *IL1B*. Interestingly, the psoriasis-associated AMPs *S100A8* and *S100A9*, are located far away from Th17 cytokines and epithelial-specific AMPs in the dendrogram. Neutrophils are rich in S100A8 and S100A9^34^ - we therefore suggest that the mRNA of these AMPs in psoriatic lesions comes not barely from keratinocytes but also from neutrophils and that their expression level can correlate with the intensity of neutrophil infiltration to the lesion. Indeed, the S100A cluster was most closely co-regulated with *IL36A* (Fig. 4a) whose excessive activity is involved in monogenic generalized pustular psoriasis, which is histologically characterized by the predominance of neutrophil infiltrations in the case of mutation in its antagonist *IL36RN* ^35–37^.

TNF-α is known for enhancing IL-17A effects on keratinocytes^13^. Our cluster analysis suggests that its specific role could be to induce *CCL2* for the recruitment of monocytes and dendritic cells to the psoriatic lesions (Fig. 4a). It is intriguing that from all the studied chemokines, *CCL27* was the only one downregulated in psoriatic skin. In epithelial cell culture, it is upregulated by IL-17A, but previous studies have convincingly shown its decrease in psoriatic lesions and suggested this as a useful biomarker for the differentiation of psoriasis from atopic dermatitis in complicated differential diagnostic cases^15^. *CCL27* is expressed in healthy skin and is recruiting CCR10 + immune cells that are important for skin homeostasis and regulation^38, 39^. Our cluster analysis corroborates previous findings that *CCL27* is downregulated by *IFNG* ^40^. Its downregulation would cease the influx of immune cells that could counterbalance inflammation in the skin, while inflammatory cells are efficiently recruited by other chemokine receptors responding to upregulated chemokines.

One of the most intriguing recent discoveries in psoriasis is the possible intimate involvement of several innate receptors in its pathogenesis^41^. Our results are in line with these findings. *IFIH1*, which encodes an RIG-I-like pattern recognition receptor melanoma differentiation-associated protein 5, recognizing dsRNA, showed elevated expression levels in psoriatic lesions. It was recently shown that AMP LL37 enables keratinocytes to produce IFN-β in response to dsRNA from dying cells^42, 43^. In our study, *CXCL10* upregulation in psoriatic skin may indicate enhanced IFN-signalling in psoriasis, and therapeutic IFNAR removal has proven to be effective in psoriatic skin inflammation treatment by UV phototherapy^44^. Moreover, AIM2, an innate receptor binding to dsDNA, was expressed at increased levels in psoriatic skin in our study, consistent with previous reports^41, 45, 46^. AIM2, when activated, recruits ASC, which is encoded by the *PYCARD* gene to form inflammasome and activate caspase 1, which cleaves pro-IL-1β to its bioactive form^47^ and induces pyroptosis^48^. Indeed, both genes – *PYCARD* and *IL1B* – showed elevated expression in psoriatic lesions. Increased levels of active caspase 1 have been revealed in lesional psoriatic epidermis by western blot^45, 49^. Using the fluorescent detection of active caspase 1, we found it in normal healthy epidermis but not in the dermis, which argues for its physiological role in epidermal maturation. Psoriatic lesions revealed increased levels of active caspase 1 per keratinocyte and also in the expense of thickened epidermis and its activation also in subepidermal dermal areas. We also confirm the recent finding that *NLRP1* and *NLRP3* inflammasomes are not differentially expressed in psoriasis^41^, although wide-spread psoriasis was shown to be associated with single-nucleotide polymorphisms in *NLRP3* ^50^. The source of the activating signal for AIM2 now remains to be identified. Keratinocytes in psoriatic skin display free cytoplasmic dsDNA^45^. The other important line of evidence comes from de Koning *et al*.^46^, who has demonstrated AIM2 staining adjacent to Munro’s abscesses using immunofluorescence. Regarding the capability of neutrophils to undergo the NETosis that releases huge amounts of immunostimulatory dsDNA, the location of AIM2 is perfect to respond to this signal. However, at this stage we can only speculate about the inflammasome type involved and the cause of its activation. Nevertheless, the finding of inflammasome and innate receptor involvement in psoriasis paves the way for the development of new effective treatment options for this serious inflammatory disease.

There is still no consensus about whether psoriasis is an autoimmune disease driven by autoantigen-specific T cells or an autoinflammatory disease provoked primarily by innate receptor signalling. Several autoantigens have been suggested, including keratin 17, LL37^42, 51^, and most recently a neolipid antigen^52^. However, the T cell response in psoriatic skin appeared to be highly polyclonal arguing against a single or a couple of autoantigens inducing the disease^31^. Among infiltrating cells there are definitely T cells: CD4 + cells mostly in dermis and CD8+ cells in epidermis^30, 53^. Our study is the first to describe the increased expression of *EOMES*, an important transcription factor for CD8+ T cell development and effector function exertion^54, 55^, in psoriatic lesions and in non-lesional skin at mRNA as well as protein level. Interestingly, *EOMES* is critical for the development of virtual memory CD8+ T cells, which emerge without TCR stimulation, are IL-15 dependent and have innate-like functions, such as the capability to rapidly produce inflammatory cytokines in the absence of antigenic recognition^17, 18, 56^. The precise role of T cells in psoriasis needs further clarification, including the possible role of virtual memory cells, their potential therapeutic modification (e.g. by blocking IL-15) and their relations with tissue-resident memory cells^57, 58^.

Psoriasis is not only a skin disease but also has many systemic features that in our study are exemplified by increased levels of several inflammatory mediators in the circulation. Moreover, the serum level of IL-17A was associated with the severity of the disease, as has also been shown in previous studies^59^, while higher IL-6 levels were characteristic of patients with psoriatic arthritis. Moreover, we found signs of excessive activation on the circulating Treg cells of psoriasis patients. CD8+ T cells had increased levels of CLA, a molecule needed for homing to skin, and significantly higher proportions of terminally differentiated and senescent CD8+ T cells that accumulate in aged persons^60^. This finding may suggest that chronic inflammation and sustained innate receptor signalling in the psoriatic organism drive premature immunosenescence in psoriasis patients, which may have important consequences on the health status of the patients. Indeed, patients with psoriasis have a tendency towards increased risk of several cancers, especially cancers of the upper aerodigestive tract, liver, respiratory tract, pancreas, urinary tract, skin squamous cell carcinoma, basal cell carcinoma and non-Hodgkin lymphoma^61^. Although photochemotherapy increases the risk of skin squamous cell carcinoma and basal cell carcinoma, the mechanisms of development of other cancers are still unknown. Immunosuppression has been implicated, whether due to treatment, chronic immune system activation or impaired immune surveillance^61^. Our data suggest that premature immunosenescence may play an important role in the inability to fight cancers. It remains to be clarified in the future whether early treatment with biologicals will stop the premature ageing of the immune system and associated co-morbidities. At least systemic anti-inflammatory treatment with methotrexate and TNF inhibitors lowers the cardiovascular risk^62^, and TNF inhibitors further reduce insulin resistance in patients with psoriasis^63^.

It needs to be acknowledged that our work has some limitations. First of all, a substantial part of our gene expression analysis confirms previously published results. Although the hierarchical cluster analysis of gene expression data provoked multiple novel hypotheses about the significance and specific roles of different gene products in psoriasis pathogenesis, the proof of these suggestions through functional experiments was out of the scope of the present study. Moreover, the functional studies confirming the innate-like phenotype of EOMES-expressing T cells purified from psoriatic skin have to be yet performed.

To conclude, in spite of the described limitations, our data are in line with studies that support the autoinflammatory pathogenesis of psoriasis that involves multiple innate receptors and innate cell types, probably including the recently described innate-like virtual memory T cell. An important aspect that needs further attention in patient care is the premature immunosenescence of CD8+ T cells and their potential involvement in impaired cancer immunosurveillance in psoriasis patients.

## Methods

### Study subjects

A case-control study was conducted at the Dermatology Clinic of Tartu University Hospital. The study was approved by the Research Ethics Committee of the University of Tartu. All the participants signed a written informed consent and all methods were performed in accordance with the relevant guidelines and regulations. Patient characteristics can be found in Supplementary Table 3.

Two skin punch biopsy samples (3–4 mm in diameter) were collected from 35 patients, one from the marginal zone of the lesional skin and another from non-sun-exposed non-lesional skin. One skin punch biopsy sample (3–4 mm in diameter) from non-sun-exposed skin was taken from each of the control subjects. The skin samples were instantly frozen in liquid nitrogen and stored at −80 °C until RNA extraction.

From all the participants, 16 ml of venous blood was collected into BD Vacutainer® CPT™ Cell Preparation Tubes with sodium heparin or with sodium citrate (BD Biosciences, Franklin Lakes, New Jersey, USA) to separate plasma and peripheral blood mononuclear cells (PBMCs). The tubes were centrifuged at 1500 g for 30 minutes. Plasma was collected and stored at −20 °C. Isolated PBMCs were washed twice with phosphate-buffered saline (PBS) and stored in freezing medium in a liquid nitrogen tank until used.

### QRT-PCR analysis

Total RNA was isolated from the skin using RNeasy Fibrous Tissue Mini Kit (Qiagen, Valencia, CA) or miRNeasy Mini Kit (Qiagen) according to the manufacturer’s instructions. For RNA extraction from the skin, the skin biopsy samples were placed in 700 µl of the QIAzol Lysis Reagent (Qiagen) and homogenized by a gentleMACS^TM^ Dissociator (Miltenyi Biotec, Heidelberg, Germany) using M tubes. The concentration and quality of the RNA were assessed with a NanoDrop ND-1000 spectrophotometer (Thermo Fisher Scientific, Wilmington, MA). cDNA was synthesized from 5000 ng of total RNA using oligo-dT and SuperScript® III Reverse Transcriptase (Life Technologies, Carlsbad, CA) according to the manufacturer’s protocols. For amplification of the PCR product SYBR® Green (Life Technologies) master mix was used. QRT-PCR analysis was carried out on ViiA^TM^ 7 Real-Time PCR system (Life Technologies) and the relative gene expression levels were calculated using the comparative Ct (∆∆*C*t) method and normalized to the expression of ACTB. Primer sequences are in Table S4.

### FAM-FLICA™ Caspase-1 Assay

Caspase-1 activation was assessed in skin cryosections with the FAM-FLICA™ Caspase-1 Assay Kit (ImmunoChemistry Technologies, Bloomington, MN) according to manufactures manual. Here briefly, 5 µm cryosections of skin biopsy specimens were fixed for 1 min with acetone and were washed twice for 5 min with PBS. Blocking was done with 10% normal goat serum (Thermo Fisher Scientific) and 0.5% bovine serum albumin (BSA) in PBS for 20 minutes. The caspase-1 activity was determined by incubating 1 hour with the FLICA Caspase-1 Reagent (FAM-YVAD-FMK) followed by washing steps. The nuclei were stained with Hoechst 33342 (2′-[4-ethoxyphenyl]-5-[4-methyl-1-piperazinyl]-2,5′-bi-1H-benzimidazole trihydrochloride trihydrate) for 10 minutes. Slides were mounted with Fluorescence Mounting medium (Dako, Santa Clara, CA) and analyzed with Leica DM5500 B microscope (Leica Microsystems, Wetzlar, Germany). Green fluorescence was quantified with the help of Fiji (ImageJ)^64^ in fixed areas of stratum spinosum. Cell nuclei were counted from the same fixed areas and the fluorescence intensity measure was divided with the number of cells in the area.

### Immunofluorescence microscopy

Immunofluorescence was performed on frozen sections of skin biopsy samples. After fixing with 4% formaldehyde, and permeabilization with 0.2% Triton-X 100 in PBS, 45 min blocking step with 1% normal goat serum and 0.5% BSA was performed. Sections were incubated overnight with antibodies to CD3 (mouse anti-human, Alexa Fluor 488-conjugated, UCHT1, Biolegend) and EOMES (rabbit polyclonal, Novus Biologicals) at 4 °C, washed in PBS, and incubated with Alexa Fluor 594-conjugated F(ab’)2-goat anti-rabbit IgG (H+L) cross-adsorbed secondary antibody (ThermoFisher Scientific, 1:500) for 1 hour. After staining with DAPI (1μg/mL) for 10 min the slides were washed once more in PBS, and covered with fluorescent mounting medium (Dako) and coverslips. Images were obtained with LSM710 confocal microscope (Zeiss, Wetzlar, Germany).

### Measurement of circulating cytokines

The level of cytokines TNF-α, IL-2, IL-1b, IL-5, IL-6, IL-8/CXCL8, IL-10, GM-CSF, IFN-γ, IL-36b, IL-33, IL-7, IL-17a, IL-17f, IL-22, IL-31, G-CSF, IL-1RA/IL-1F3, CXCL10/IP-10 and Lipocalin-2/NGAL in heparin treated plasma was measured by the xMAP technology on Luminex 200 (Luminex Corporation, Austin, TX). The Milliplex MAP multiplex assay was conducted in a 96-well microplate format according to the manufacturer’s instructions (Millipore, Billerica, MA).

### Flow cytometry

Surface marker expression on PBMC was assessed by flow cytometry. Cells were stained in flow cytometry staining buffer (PBS with 0.5% bovine serum albumin and 0.1% sodium azide) for 20 min at 4 °C with various antibodies as indicated. T cells were stained with CCR7, CD127, CD25, CD28, CD3, CD31, CD4, CD45RA, CD8, CLA, HLA-DR and TIGIT monoclonal antibodies described in Supplementary Table S5.

Viable cells were distinguished using LIVE/DEAD Fixable Dead Cell Stain Kit (Thermo Fisher Scientific) according to the manufacturer’s instructions. Populations of interest were gated according to appropriate “fluorescence minus one” controls. Samples were acquired on a LSRFortessa flow cytometer (BD Biosciences, Franklin Lakes, NJ) and then analyzed using FACSDiva version 6 (BD Biosciences) software and FCS Express 5 (De Novo Software, Glendale, CA). Optical detectors configuration used to acquire samples is shown in Supplementary Table S6. For compensation matrix see Supplementary Table S7. Gating strategy is shown in Supplementary Fig. S6.

### Biostatistics and data visualization

#### CT values preprocessing

In case when two or more Ct values from QRT-PCR per one patient were undetermined, i.e. fail to reach the prespecified minimum signal intensity threshold (i.e 40 cycles)^65, 66^, they were imputed using expectation-maximization algorithm described in McCall *et al*.^66^, implemented in R package *nondetects* ver. 2.2.0 available from Bioconductor^67^.

#### Filtering of the reference gene CT values based on interquartile rate

In order to ensure expression stability of the reference gene, its Ct values were filtered based on interquartile range (IQR). Observations that fall below Q1–1.5(IQR) or above Q3 + 1.5(IQR) were considered as outliers.

#### Manufacturer’s protocol

ΔCt, Δ ΔCt values, corresponding standard deviations for all the target genes and the reference genes, and the standard errors of measurement were calculated and filtered based on the errors of measurement <20% according to the manufacturer’s protocol. Gene expression was calculated as 2^−Δ ΔCt^ and then log2-transformed for the further analysis.

#### Clustering and PCA

Hierarchical cluster analysis was performed by using Euclidean distance metric and Ward’s linkage method implemented in *amap* R package ver. 0.8–14^68^. Clusters that are strongly supported by the data were identified using multiscale bootstrap resampling approach described in Suzuki & Shimodaira^69^ and implemented in *pvclust* R package ver. 2.0–0^69^. Clusters were selected using default package parameters and *p-value* cut-off 0.05.

Missing values in the preprocessed dataset of the expression values in skin samples were imputed using the k-nearest neighbour (KNN, k = 4) method implemented in *Impute* R package ver. 1.46.0 from Bioconductor^70^. PCA of the skin samples was performed on the KNN-imputed expression values. The first three principal components covered correspondingly 47%, 12% and 7% (0.4637 0.1523 0.0626) of the variance in the data.

#### Differential expression

Differential expression analysis in skin samples was performed using Linear Models for Microarray and RNA-Seq Data *(limma)* R package ver. 3.28.21 from Bioconductor^71^. Genes with statistically significant difference in the expression levels were selected based on logFC ≥ 1 and *p-value* ≤ 0.05 corrected for multiple testing using FDR method.

#### Analysis of the cytokines’ concentration levels in plasma

Plasma samples were quantile-normalized^72, 73^ and log2-transformed. Wilcoxon signed-rank test^74^ was used to identify cytokines with statistically significant differences in concentration levels in psoriasis patients and control group. P-values were adjusted using false discovery rate correction for multiple comparisons. Cytokines with statistically significant changes in psoriasis patients and control group were selected based on *p-value* ≤ 0.05.

#### Phenotype comparison

Comparisons of the selected phenotypes in skin and plasma samples were performed using multi-factor ANOVA, followed by Tukey’s multiple comparison test to find the groups that are significantly different from each other. Results then were filtered based on the adjusted *p-value* ≤ 0.05. Phenotype comparison was carried out for the differentially expressed genes in skin and plasma cytokines that showed statistically significant changes between psoriasis patients and control group.

#### Comparison of T cell subpopulations among PBMCs isolated from psoriasis patients and control individuals

Wilcoxon signed-rank test was used to identify statistically significant differences in the T cell subpopulations among PBMCs isolated from psoriasis patients and control group. P-values were adjusted using false discovery rate correction for multiple comparisons. Groups with statistically significant differences in psoriasis patients and control group were selected based on *p-*value ≤ 0.05.

#### Data visualization

Data were visualized with box plots and heatmaps using R-packages *ggplot2* ver. 2_2.1.0^75^, gridExtra ver. 2.2.1^76^ and *pheatmap* ver. 1.0.8^77^.

## Electronic supplementary material

Supplemental material
