# Supplementary material for: Signs of innate immune activation and premature immunosenescence in psoriasis patients
Source: Sci Rep. 2017 Aug 8;7:7553. doi: 10.1038/s41598-017-07975-2 (PMC5548718; doi:10.1038/s41598-017-07975-2)
Supplement: Supplementary file 1 — Supplemental material [file 41598_2017_7975_MOESM1_ESM.pdf]

SUPPLEMENTARY MATERIAL

Signs of innate immune activation and premature immunosenescence in psoriasis patients

Liisi Šahmatova[1, 2#], Elena Sügis[3, 4#], Marina Šunina[5#], Helen Hermann[5], Ele Prans[5], Maire Pihlap[5], Kristi Abram[2], Ana Rebane[5], Hedi Peterson[3, 4], Pärt Peterson[5], Külli Kingo[1, 2], Kai Kisand[5*]

[1] Department of Dermatology, University of Tartu, Estonia

[2] Dermatology Clinic, Tartu University Hospital, Estonia

[3] Institute of Computer Science, University of Tartu, Estonia

[4] Quretec Ltd, Tartu, Estonia

[5] Institute of Biomedicine and Translational Medicine, University of Tartu, Estonia

[#]These authors contributed equally to this work

SUPPLEMENTARY TABLES

Table S1. Gene expression data and group comparisons for skin biopsy samples

Gene name	C Median (Q1-Q3)	L Median (Q1-Q3)	NL Median (Q1-Q3)	logFC LvsC	P LvsC	logFC NLvsC	P NLvsC	logFC LvsNL	P LvsNL	N_C	N_L	N_{NL}
AIM2	2.93 (2.2-5.1)	9.39 (5.09-14.73)	3.91 (3.2-4.7)	1.67	8.27E-06	0.25	5.58E-01	1.42	1.83E-05	22	30	23
CASP1	23.91 (18.84-31.89)	25.55 (18.01-28.55)	20.2 (16.68-23.75)	-0.04	8.36E-01	-0.3	1.04E-01	0.26	1.24E-01	23	30	28
CCL2	10.22 (7.95-14.8)	26.22 (19.48-35.03)	13.18 (9.37-19.29)	1.26	2.75E-07	0.42	7.76E-02	0.84	2.65E-05	23	33	31
CCL20	0.03 (0.03-0.05)	0.82 (0.5-1.27)	0.1 (0.04-0.21)	4.25	7.04E-15	1.53	2.24E-02	2.72	1.54E-07	17	31	27
CCL27	158.98 (102.02-189.54)	33.21 (21.08-56.08)	147.61 (109.77-206.19)	-2.12	9.63E-11	0	9.95E-01	-2.12	1.12E-15	21	33	31
CCL5	5.69 (4.51-7.41)	16.06 (6.12-27.72)	9.67 (6.84-13.03)	1.15	2.08E-04	0.76	1.26E-02	0.39	1.89E-01	23	33	31
CTLA4	6.31 (3.02-7.12)	29.68 (15.15-95.26)	11.2 (6.88-13.92)	3	3.26E-10	1.33	2.74E-03	1.67	1.35E-05	21	32	28
CXCL1	0.00059 (0.00043-0.0015)	0.02 (0.0088-0.03)	0.0013 (0.0007-0.0022)	4.54	7.29E-16	0.91	4.92E-02	3.63	6.37E-14	20	32	27
CXCL10	2.07 (1.04-3.29)	19.14 (13.11-25.97)	5.39 (3.12-11.14)	3.14	8.75E-15	1.53	1.96E-03	1.62	2.32E-06	19	32	29
CXCL2	0.0075	0.03	0.0097	2.05	9.42E-09	0.32	5.32E-01	1.74	2.14E-08	18	31	25

	(0.0042-0.01)	(0.02-0.04)	(0.005-0.02)									
CXCL8	0.0014<br>(0.00067-0.0027)	0.1<br>(0.06-0.3)	0.0017<br>(0.00088-0.0021)	6.56	5.77E-12	0.71	6.03E-01	5.85	4.92E-10	11	32	14
DEFB1	34.58<br>(26.65-132.64)	23.11<br>(16.42-32.44)	26.26<br>(19.92-37.08)	-1.15	6.38E-04	-1.12	2.20E-02	-0.04	8.88E-01	23	33	31
EOMES	0.75<br>(0.6-2.19)	3.1<br>(1.71-4.83)	2.05<br>(1.83-4.35)	1.46	7.63E-04	1.27	3.66E-03	0.19	5.82E-01	15	31	27
FOXP3	18.26<br>(8.08-22.56)	65.03<br>(33.56-115.13)	16.7<br>(12.16-23.56)	2.04	5.72E-08	0.24	5.75E-01	1.81	6.29E-11	22	32	31
IFIH1	7.58<br>(5.68-9.95)	16.82<br>(12.97-35.05)	11.63<br>(6.68-14.42)	1.28	1.22E-04	0.59	3.63E-02	0.69	3.65E-02	23	32	29
IFNAR1	1.1<br>(0.7-1.29)	0.84<br>(0.55-1.12)	0.92<br>(0.75-1.1)	-0.3	2.47E-01	-0.08	7.63E-01	-0.21	2.45E-01	23	33	31
IFNGR	0.86<br>(0.54-1.07)	1.24<br>(0.92-1.87)	0.82<br>(0.71-1.12)	0.64	1.50E-02	0.2	5.11E-01	0.44	2.87E-02	23	33	31
IL10	0.33<br>(0.24-0.45)	1.06<br>(0.72-1.74)	0.69<br>(0.5-0.86)	1.86	9.56E-07	1.03	1.81E-03	0.83	2.20E-03	21	25	26
IL17A	0.000018<br>(0.0000014-0.0000023)	0.58<br>(0.34-0.78)	0.0000027<br>(0.0000017-0.0000034)	17.17	2.26E-28	1.05	5.91E-01	16.12	6.99E-24	18	31	20
IL17F	0.006<br>(0.0043-0.0087)	1.76<br>(0.75-2.99)	0.01<br>(0.0068-0.02)	8.09	5.52E-27	1.35	3.82E-02	6.74	4.92E-20	16	27	24
IL1B	0.11<br>(0.04-0.27)	0.62<br>(0.45-1.43)	0.07<br>(0.05-0.12)	2.5	1.61E-05	-0.46	5.58E-01	2.96	2.20E-08	19	30	25
IL36	46.76	2250.97	118.19	5.79	4.41E-28	1.9	1.71E-05	3.90	2.21E-20	23	33	31

	(25.29-59.53)	(1615.39-2704.23)	(70.77-175.2)									
IL1RN	41.07 (23.5-53.81)	107.98 (87.06-149.15)	60.49 (51.95-73.47)	1.74	6.03E-09	0.93	4.80E-04	0.80	8.02E-05	23	33	31
IL20RA	9.15 (5.43-12.37)	5.64 (3.84-8.93)	6.92 (5.1-9.02)	-0.22	6.41E-01	0.18	7.63E-01	-0.40	1.16E-01	23	29	31
IL22	0.00084 (0.00071-0.0014)	10 (4.42-16.59)	0.0063 (0.004-0.0083)	13.01	8.53E-32	3.19	2.69E-04	9.81	1.68E-20	17	23	22
IL22RA1	11.16 (4.31-12.76)	21.51 (13.96-26.81)	13.34 (11.27-16.4)	1.7	1.11E-05	1.06	9.25E-03	0.64	4.60E-04	23	32	31
IL22RA2	15.49 (4.39-25.85)	26.34 (18.38-38.3)	12.35 (7.52-26.56)	0.95	3.61E-02	0.22	7.63E-01	0.73	7.36E-02	17	25	18
IL26	1.21 (0.81-1.82)	8.59 (4.29-15.98)	1.36 (0.72-2.77)	2.77	1.01E-04	0.42	7.56E-01	2.36	2.73E-04	5	22	8
INFG	44.3 (29.84-78.76)	371.27 (297.69-563)	60.69 (38.89-73.03)	3.17	3.56E-08	0.25	5.91E-01	2.92	1.40E-09	17	13	19
KLRK1	24.43 (18.73-37.3)	33.17 (17.43-44.29)	30.43 (23.5-47.92)	0.06	8.50E-01	0.37	3.00E-01	-0.31	3.05E-01	22	30	28
LCN2	0.0048 (0.0024-0.0086)	0.19 (0.13-0.28)	0.0092 (0.004-0.03)	4.95	6.76E-16	1.09	1.27E-01	3.86	1.25E-07	22	27	16
MICB	0.12 (0.05-0.15)	0.18 (0.12-0.22)	0.14 (0.11-0.17)	0.96	1.35E-02	0.83	9.25E-03	0.13	6.57E-01	23	33	30
NLRP1	13.39 (7.47-15.33)	8.06 (5.49-10.88)	13.16 (9.98-15.45)	-0.36	1.97E-01	0.2	5.32E-01	-0.56	1.76E-02	23	30	30

NLRP3	2.64 (1.57-3.48)	2.91 (1.7-4.32)	1.95 (1.1-3.37)	0.24	6.41E-01	-0.19	7.63E-01	0.43	3.02E-01	11	18	17
PI3	0.14 (0.05-0.25)	75.13 (58.46-102.04)	0.23 (0.09-0.42)	9.08	8.53E-32	1.34	7.76E-02	7.74	1.18E-21	23	33	31
PYCARD	33.4 (28.4-56.46)	64.04 (56.81-96.65)	38.55 (29.33-43.64)	0.93	1.11E-05	0.03	8.83E-01	0.90	1.64E-06	23	33	29
REG3A	106.74 (86.43-145.22)	38.49 (21.66-74.32)	100.36 (85.91-117.23)	-1.53	2.93E-02	0.16	8.10E-01	-1.70	4.01E-02	7	8	7
S100A8	6.51 (1.78-25.07)	939.45 (731.64-1092.69)	8.09 (1.93-14.17)	6.99	1.37E-21	0.27	7.87E-01	6.72	2.22E-20	23	33	31
S100A9	2.74 (1.42-13.32)	1532.5 (936.2-1669.27)	8.76 (2.62-23.96)	8.29	6.67E-27	1.26	1.96E-01	7.03	7.82E-19	23	33	30
TNF	14.45 (6.36-17.85)	24.3 (16.1-32.37)	18.16 (13.49-21.75)	1.14	1.22E-04	0.84	9.70E-03	0.29	1.38E-01	23	32	31
TRGC1	46.74 (28.9-60.13)	58.79 (45.63-128.56)	40.5 (30.4-66.04)	0.51	2.27E-01	-0.08	8.47E-01	0.58	1.58E-01	20	29	26
WIPI1	24 (21.71-37.89)	24.96 (19.36-27.96)	24.39 (20.05-29.83)	-0.26	1.85E-01	-0.17	4.22E-01	-0.09	5.55E-01	23	33	31

C - control skin, L - lesional skin, NL - nonlesional skin, FC - fold change, N_C –number of control samples, N_L –number of lesional skin samples, N_{NL} –number of nonlesional skin samples

Table S2. The percentages and mean fluorescence indexes of key T cell subpopulations and cell surface markers as studied with flow cytometry

Cell subset	Psoriasis patients		Healthy controls		P-value
	% of cell subset within corresponding lymphocytes				
	Mean	Range	Mean	Range	
CD4+ T cells (within CD3+ T cells)	56.7	41.3 – 73.1	64.5	45.3 – 78.2	ns
Naive CD4+ T cells (within CD4+ T cells)	46.9	33.3 – 69.6	50.1	24.4 - 68.4	ns
RTE (CD31+ naive CD4+ T cells within CD4+ cells)	27.6	13.8 – 53.5	30.2	14.2 – 53.9	ns
CM CD4+ T cells (within CD4+ T cells)	37.2	15.0 – 50.4	33.3	19.4 – 52.0	ns
EM CD4+ T cells (within CD4+ T cells)	14.2	3.8 – 23.7	13.8	8.3 – 24.4	ns
CD4+ T_{EMRA} (within CD4+ T cells)	1.7	0.1 – 7.0	2.8	0.3 – 16.6	ns
HLA-DR+ CD4+ T cells (within CD4+ T cells)	1.2	0.6 – 2.4	0.7	0.3 – 1.3	ns
CD8+ T cells (within CD3+ T cells)	36.7	21.1 – 51.1	26.3	9.7 – 45.7	0.0347
Naive CD8+ T cells (within CD8+ T cells)	34.4	10.9 – 70.9	36.6	14.4 – 62.3	ns
RTE (CD31+ naive CD8+ T cells within CD8+ cells)	31.1	7.2 – 68.3	34.5	12.7 – 60.6	ns
CM CD8+ T cells (within CD8+ T cells)	7.5	0.9 – 17.0	10.2	4.0 – 18.5	ns
EM CD8+ T cells (within CD8+ T cells)	31.6	16.0 – 48.1	40.6	18.4 – 63.5	ns
CD8+ T_{EMRA} (within CD8+ T cells)	26.5	3.8 – 55.8	12.7	3.7 – 36.7	0.0443
CD28- CD8+ T_{EMRA} (within CD8+ T cells)	23.0	2.6 – 53.7	11.1	1.9 – 35.8	0.0443
Tregs (CD25+CD127-) (within CD3+ T cells)	3.0	0.9 – 4.9	2.5	1.3 – 3.5	ns
HLA-DR+ Tregs (within Tregs)	14.0	4.5 – 22.9	8.7	4.9 – 16.0	0.0347
HLA-DR+ CD4+ T cells	1.2	0.6 – 2.4	0.7	0.3 – 1.3	ns

(within CD4+ T cells)					
	MFI of corresponding lymphocyte subset				
	Mean	Range	Mean	Range	
Treg CM HLA-DR MFI	255	165 – 358	183	135 – 264	0.0291
Naive CD8+ T cells CLA MFI	241	137 – 651	226	164 – 330	ns
CM CD8+ T cells CLA MFI	4572	2796 – 11154	4008	2258 – 6012	ns
EM CD8+ T cells CLA MFI	3772	1623 – 5581	2250	1342 – 4747	0.0443
CD8+ T_{EMRA} CLA MFI	750	178 – 2837	658	302 – 2053	ns

CLA – cutaneous leukocyte antigen, CM – central memory, EF – effector memory, MFI – median fluorescence intensity, RTE – recent thymic emigrant, T_{EMRA} – effector memory T cell with CD45RA expression

Table S3. Characteristics of participants.

Psoriasis patients were collected from the outpatient or inpatient department of the Dermatology Clinic. None of the patients had received any treatment for their psoriasis for at least a month before recruitment. Control subjects were recruited from among healthcare personnel, medical students and patients who turned to the dermatological outpatient clinic for surgical excision of nevus. None of the control subjects had any chronic skin disease history or psoriasis in their family.

**Characteristic**	**Psoriasis patients**	**Control subjects**
Total number of participants	33 (41)[#]	23
Number of males/females	25/10 (31/10)[#]	8/16
Age range (years)	20-63	24-57
PASI score range	2-44	
Number of patients with psoriasis arthritis	10 (12)	
Number of patients with nail involvement	27 (31)	
Number of patients with early onset psoriasis/late onset psoriasis[*]	29/6 (33/6)	
Positive family history of psoriasis	19	

[*]Early onset psoriasis is defined as a disease with onset before the age of 40 years and late onset psoriasis is defined as a disease with onset at or after the age of 40 years

[#] in brackets the total number of recruited patients is shown, the number before the brackets stands for patients with two parallel skin biopsy specimens analysed.

Table S4. Primer sequences.

Gene	Forward primer	Reverse primer
ACTB – actin beta	CTGGAACGGTGAAGGTGACA	CGGCCACATTGTGAACTTTG
AIM2 – absent in melanoma 2	TAAGTCAAGCTGAAATGAGTCCTG	TTCTGTTCAGGCTTAACATGAGG
CASP1 – caspase 1	AAAGTCGGCAGAGATTTATCCA	GATGTCAACCTCAGCTCCAG
CCL2 – C-C motif chemokine ligand 2	AAGCTGTGATCTTCAAGACC	GTTCAAGTCTTCGGAGTTTGG
CCL5 - C-C motif chemokine ligand 5	CCATATTCCTCGGACACCAC	TTTCGGGTGACAAAGACGAC
CCL20 - C-C motif chemokine ligand 20	ATCAGAAGCAAGCAACTTTGAC	TGATAGCATTGATGTCACAGCC
CCL27 - C-C motif chemokine ligand 27	AGAGAAGGAAGAGTCTAGGCTG	GTAGGAATGCTGCTGTAGGG
CTLA4 – cytotoxic T-lymphocyte associated protein 4	TGAACCTGGCTACCAGGACC	CCACGTGCATTGCTTTGC
CXCL1 – C-X-C motif chemokine ligand 1	ATGCTGAACAGTGACAAATCC	CTCAAACACATTAGGCACAATCC
CXCL2 – C-X-C motif chemokine ligand 2	CCAAACCGAAGTCATAGCCACAC	GCCACCAATAAGCTTCCTCCT
CXCL8 – C-X-C motif chemokine ligand 8	GAGAGTGATTGAGAGTGGAC	GAATTCTCAGCCCTCTTCAAA
CXCL10 – C-X-C motif chemokine ligand 10	TTCCTGCAAGCCAATTTTGT	TTCTTGATGGCCTTCGATTC
EOMES - eomesodermin	GACAACTATGATTCATCCCATCAG	GGCTTGAGGTAAAGTGTTGAC
FOXP3 – forkhead box 3	TGGAGAAGGAGAAGCTGAGTGC	CCCTTGTCGGATGATGCCA
IFIH1 – interferon induced with helicase C domain 1	ATGGAGCAATATACTAGGACTG	TTGTTCATTCTGTGTCATGG
IFNAR1 - Interferon alpha and beta receptor subunit 1	CCATCCAAAGCCCACATAACACT	CGCAAAGCTCAGATTGGTCCT

IFNG – interferon gamma	TTCGGTAACTGACTTGAATGTCCA	TTTCGCTTCCCTGTTTTAGCTG
IFNGR – interferon gamma receptor 1	GCTCGTCTCCATTTACAAAAACTGA	GGAAAAATTGGACCACCTAAACTG
IL10 – interleukin 10	TTCCCAGGCAACCTGCCTAA	TGTCCAGCTGATCCTTCATTTGA
IL17A – interleukin 17A	ACCGCAATGAGGACCCTGAGA	TCCACGTTCCCATCAGCGT
IL17F – interleukin 17F	GGAATTACACTGTCACTTGG	GATGTCTTCCTTTCCTTGAG
IL1B – interleukin 1 beta	AAACAGATGAAGTGCTCCTTCC	CATCTTCCTCAGCTTGTCCA
IL36A – interleukin 36, alpha	GCCGTCTATCAATCAATGTGTA	ACTGTCACTTCGTGGAACTG
IL1RN – interleukin receptor antagonist	AAGATAGATGTGGTACCCATTGAG	TGATGTTAACTGCCTCCAGC
IL20RA – interleukin 20 receptor subunit alpha	GAACCTACTGTGATCTTTCTGCT	GTGGGCCAATTTGTGTTTCTAA
IL22 – interleukin 22	AGCACATGTCATATTGAAGG	GTTCTCCAATTGCTTTGATCTC
IL22RA1 – interleukin 22 receptor subunit alpha 1	ATCGAGTATAAGACGTACGGAG	CATAGTAGAGCTCCGTGAGG
IL22RA2 - interleukin 22 receptor subunit alpha 2	TGGTGTAGCAGGAACTCAGTC	TGCTGTTGCCAGTAAGTGCC
IL26 – interleukin 26	ATTTCAAGAACAGCTTCTGTCC	AAGCACAGGAAATACAGTGG
KLRK1 – killer cell lektin like receptor K1	TATACAGCAAAGAGGACCAGGA	TATTGTTAGTAGGTTGGGTGAGAG
LCN2 – lipocalin 2	GGCAACATTAAGAGTTACCCTGGA	GTTCTCCTTTAGTTCCGAAGTCAG
MICB – MHC class I polypeptide-related sequence B	CTATGACAGGCAGAAACGCA	GAATGCAAGCCTCCTTTCTG
NLRP1 – NLR family pyrin domain containing 1	ACTTGTACCGAGTTCACTTCC	CTGTGCTGTGGGTTGATCTC
NLRP3 - – NLR family pyrin domain containing 3	ACATGCCCAAGGAGGAAGAG	GGCTGTTCACCAATCCATGAG
PI3- peptidase inhibitor 3	TCACGGGAGTTCCTGTTAAAGGTC	CTTTGACTGGCTCTTGCGCT

PYCARD – PYD and CARD domain containing	CTGGTCAGCTTCTACCTGGA	CTATAAAGTGCAGGCCTGGC
S100A8 – S100 calcium binding protein A8	GTCTTTCAGAAGACCTGGTG	CGGTCTCTAGCAATTTCTTCAG
S100A9 - S100 calcium binding protein A9	AACACCTTCCACCAATACTC	TTCATTCTTATTCTCCTTCTTGAG
TNF – tumor necrosis factor	CCCATGTTGTAGCAAACCCT	AGGACCTGGGAGTAGATGAG
TRGC1 – T cell receptor gamma constant 1	GGACAAAGAACACAGATGTATCGT	GCAGCAGTAGTGTATCATTTGCA
WIPI1 – WD repeat domain, phosohoinositide interacting 1	TTTCCTCCTATAATCTTGTGCCGT	ACTCCATTCTTCCGCCTTCC

Table S5. Used antibodies.

Antibody and dye	Manufacturer	Clone
APC-Cy7 Streptavidin	BioLegend	-
CD127 Biotin	eBioscience	eBioRDR5
CD197 (CCR7) PE-CF594	BD Biosciences	150503
CD25 Brilliant Violet 711™	BioLegend	BC96
CD28 PE-Cy5	BioLegend	CD28.2
CD3 PerCP-Cy5.5	BioLegend	HIT3a
CD31 APC	BioLegend	WM59
CD4 Alexa Fluor® 700	BioLegend	OKT4
CD45RA PE-Cy7	BioLegend	HI100
CD8a Brilliant Violet 605™	BioLegend	RPA-T8
CLA FITC	BioLegend	HECA-452
HLA-DR Brilliant Violet 510™	BioLegend	L243
LIVE/DEAD® Fixable Blue Dead Cell Stain	Thermo Fisher Scientific	-
TIGIT PE	eBioscience	MBSA43

Table S6. Optical detectors configuration of flow cytometer.

Laser	PMT	LP Mirror	BP Filter	Intended Dye
488-nm blue laser	A	685	710/50	PerCP-Cy5.5
	B	505	530/30	FITC
	C		488/10	Side scatter (SSC)
405-nm violet laser	A	670	710/40	Brilliant Violet 711™
	B	630	670/30	-
	C	600	610/20	Brilliant Violet 605™
	D	535	540/30	-
	E	505	525/50	Brilliant Violet 510™
	F	-	440/40	-
561-nm YG laser	A	750	780/60	PE-Cy7
	B	685	710/50	-
	C	635	670/30	PE-Cy5
	D	600	610/20	PE-CF594
	E	-	586/15	PE
355-nm UV laser	A	505	530/30	-
	B	-	450/50	LIVE/DEAD®
640-nm red laser	A	750	780/60	APC-Cy7
	B	690	730/45	Alexa Fluor® 700
	C	-	670/14	APC

Table S7. Spillover matrix (values in %).

	1. FITC-A	2. PerCP-Cy5-5-A	3. Brilliant Violet 510-A	4. Brilliant Violet 605-A	5. Brilliant Violet 711-A	6. LIVE/DEAD-A	7. APC-A	8. Alexa Fluor 700-A	9. APC-Cy7-A	10. PE-A	11. PE-CF594-A	12. PE-Cy5-A	13. PE-Cy7-A
1.	100	2	4	1	0	0	0	0	0	0	0	0	0
2.	0	100	0	0	50	0	14	32	6	0	0	16	6
3.	0	0	100	46	15	1	0	0	0	0	0	0	0
4.	0	3	1	100	40	0	0	0	0	34	58	14	2
5.	0	14	0	0	100	0	2	50	9	0	0	0	1
6.	1	0	2	0	0	100	0	0	0	0	0	0	0
7.	0	0	0	0	2	0	100	35	5	0	0	12	1
8.	0	2	0	0	4	0	1	100	15	0	0	0	2
9.	0	0	0	0	0	0	9	17	100	0	0	1	19
10.	0	3	0	1	0	0	0	0	0	100	34	4	0
11.	0	15	0	3	1	0	0	0	0	22	100	19	2
12.	0	70	0	0	5	0	81	32	5	3	1	100	11
13.	0	2	0	0	0	0	0	1	10	4	2	0	100

SUPPLEMENTAL FIGURES

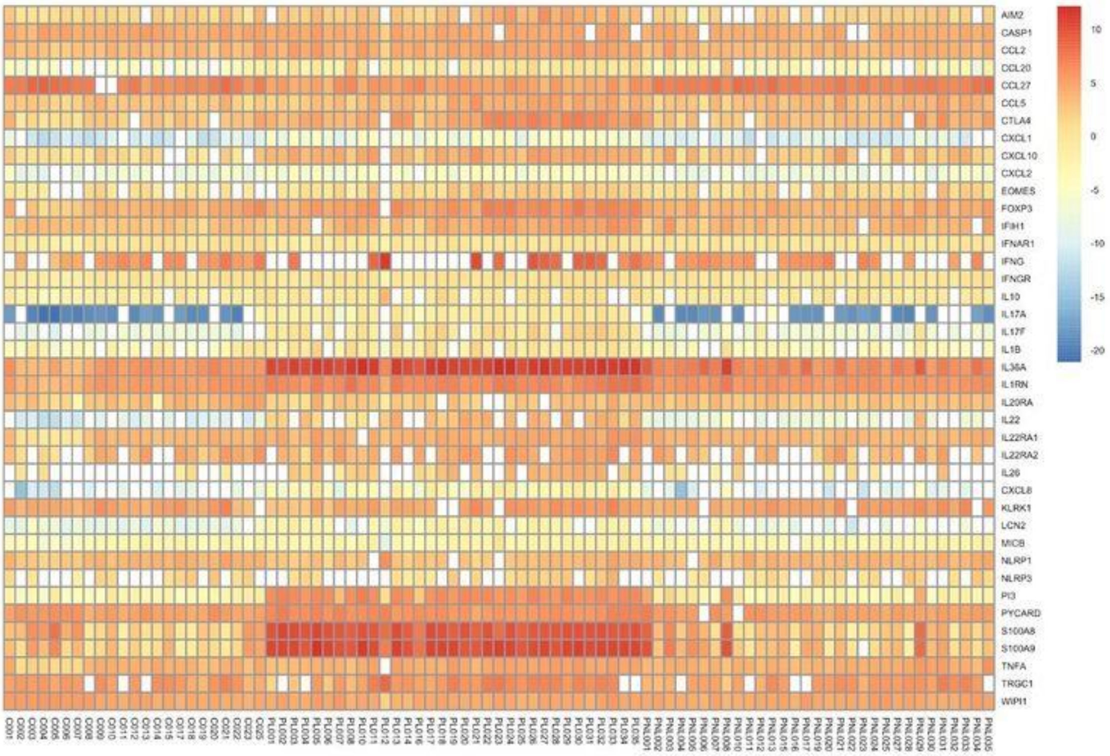

Figure S1. Heatmap of log2 transformed gene expression data.

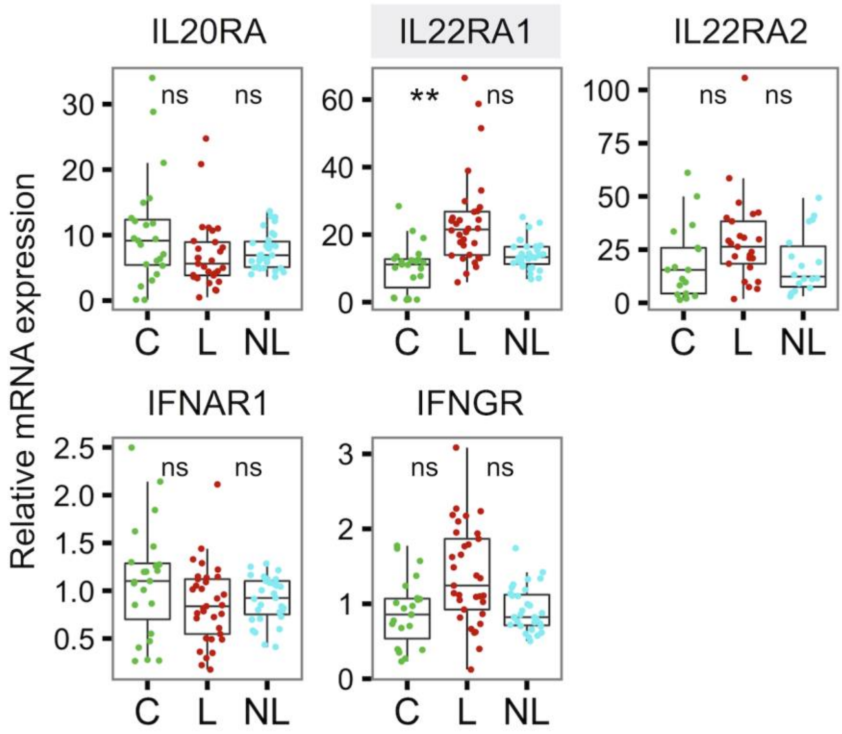

Figure S2. Gene expression of several cytokine receptors in skin biopsy samples. QRT-PCR was used to quantify the expression of the receptor genes in skin biopsy samples obtained from lesional (L) and nonlesional (NL) skin of psoriasis patients as well as from age-matched control subjects (C). The box-and-whisker plots depict median (central line), interquartile range (IQR, Q1-Q3, box), Q3 + 1.5 x IQR (upper whisker) and Q1 – 1.5 x IQR (lower whisker). Stars above the groups C and NL depict their significance level from L samples. ** $P<0.001$. Gray shading behind the gene name indicates to statistically significant differences between NL and C biopsy samples (for IL22RA1 $P=0.0092$)

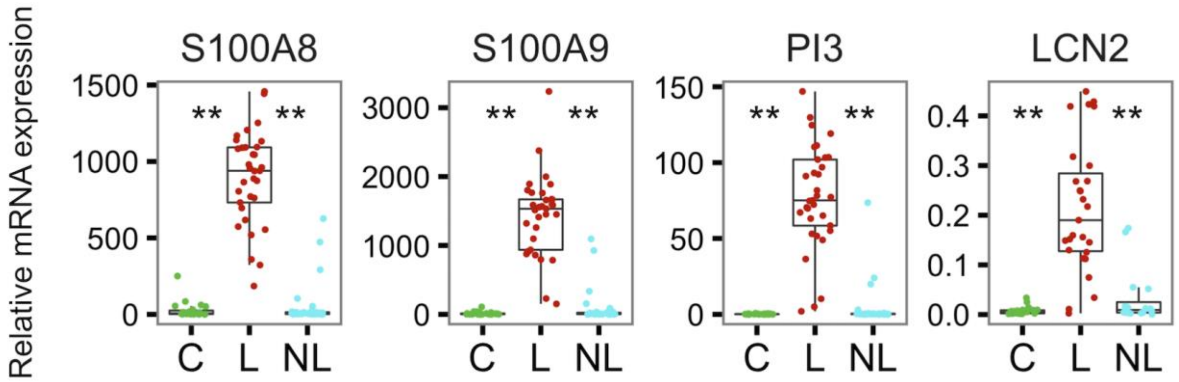

Figure S3. Gene expression of several AMPs in skin biopsy samples. QRT-PCR was used to quantify the expression of genes encoding several AMPs in skin biopsy samples obtained from lesional (L) and nonlesional (NL) skin of psoriasis patients as well as from age-matched control subjects (C). The box-and-whisker plots depict median (central line), interquartile range (IQR, Q1-Q3, box), Q3 + 1.5 x IQR (upper whisker) and Q1 – 1.5 x IQR (lower whisker). Stars above the groups C and NL depict their significance level from L samples. ** $P<0.001$.

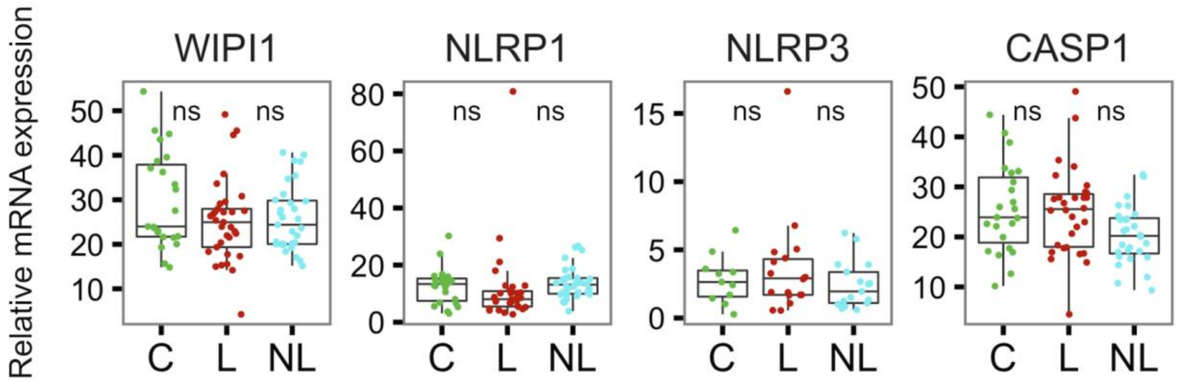

Figure S4. Gene expression data of autophagy and inflammasome related genes in skin biopsy samples. QRT-PCR was used to quantify the expression of selected genes in skin biopsy samples obtained from lesional (L) and nonlesional (NL) skin of psoriasis patients as well as from age-matched control individuals (C). The box-and-whisker plots depict median (central line), interquartile range (IQR, Q1-Q3, box), Q3 + 1.5 x IQR (upper whisker) and Q1 – 1.5 x IQR (lower whisker).

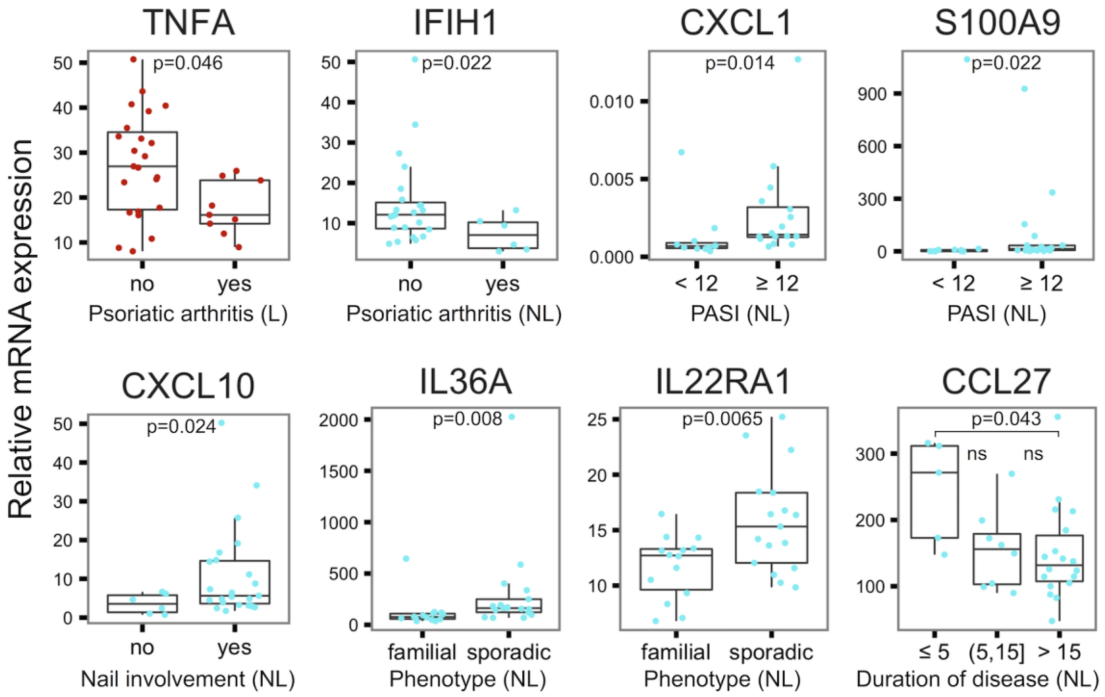

Figure S5. Gene expression data in skin biopsy samples derived from psoriasis patients with different phenotypic features. Red dots represent samples from psoriatic lesions (L) and blue dots samples from non-lesional (NL) skin of psoriasis patients. The box-and-whisker plots depict median (central line), interquartile range (IQR, Q1-Q3, box), Q3 + 1.5 x IQR (upper whisker) and Q1 – 1.5 x IQR (lower whisker).

Figure S6. Gating strategy for T cell subpopulations in PBMC samples

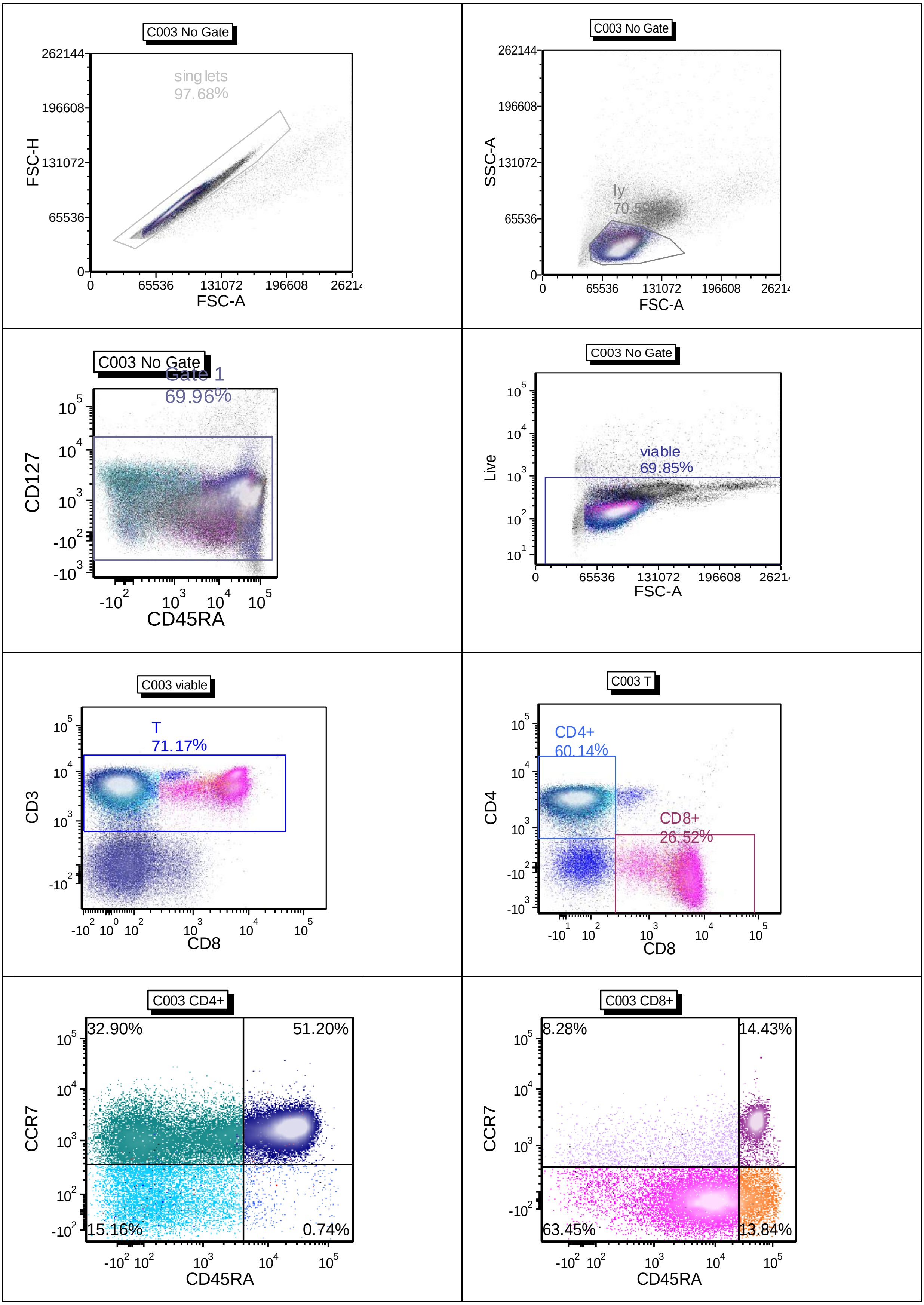

Upper left – CM, upper right – naïve, lower left - EM, lower right - T_{EMRA}	Upper left – CM, upper right – naïve, lower left - EM, lower right - T_{EMRA}
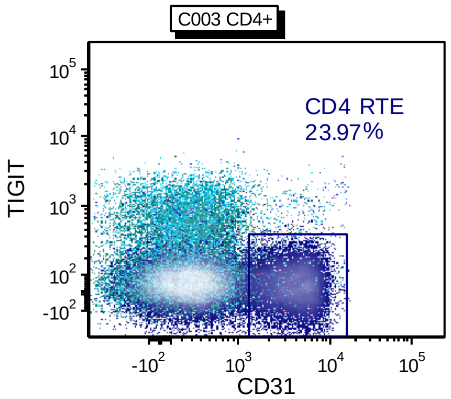	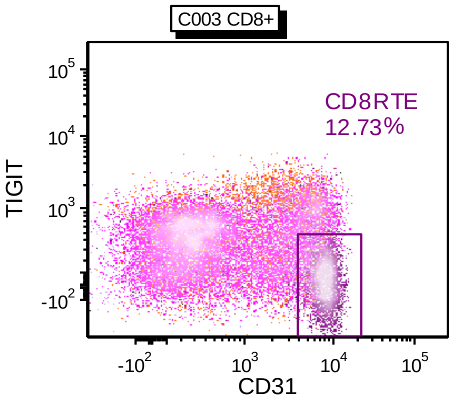
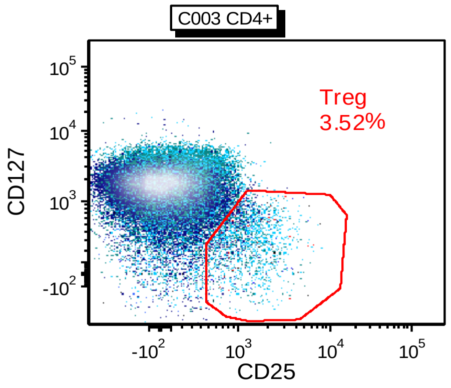	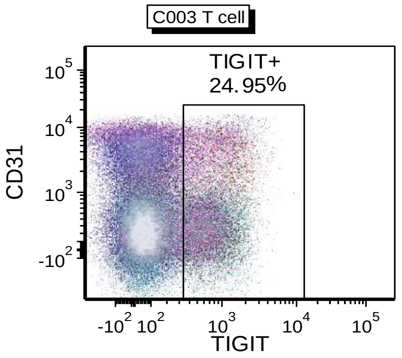
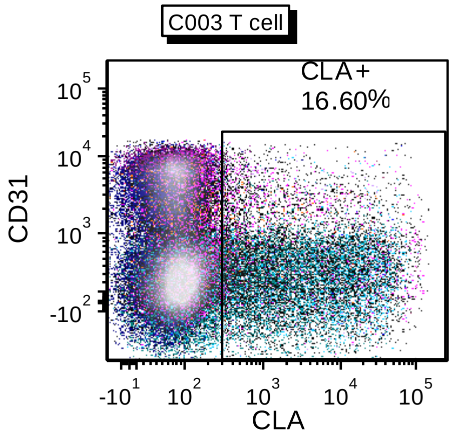	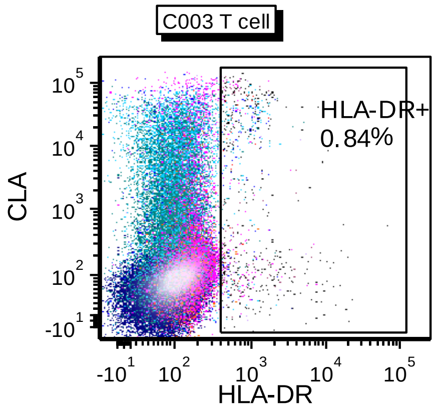

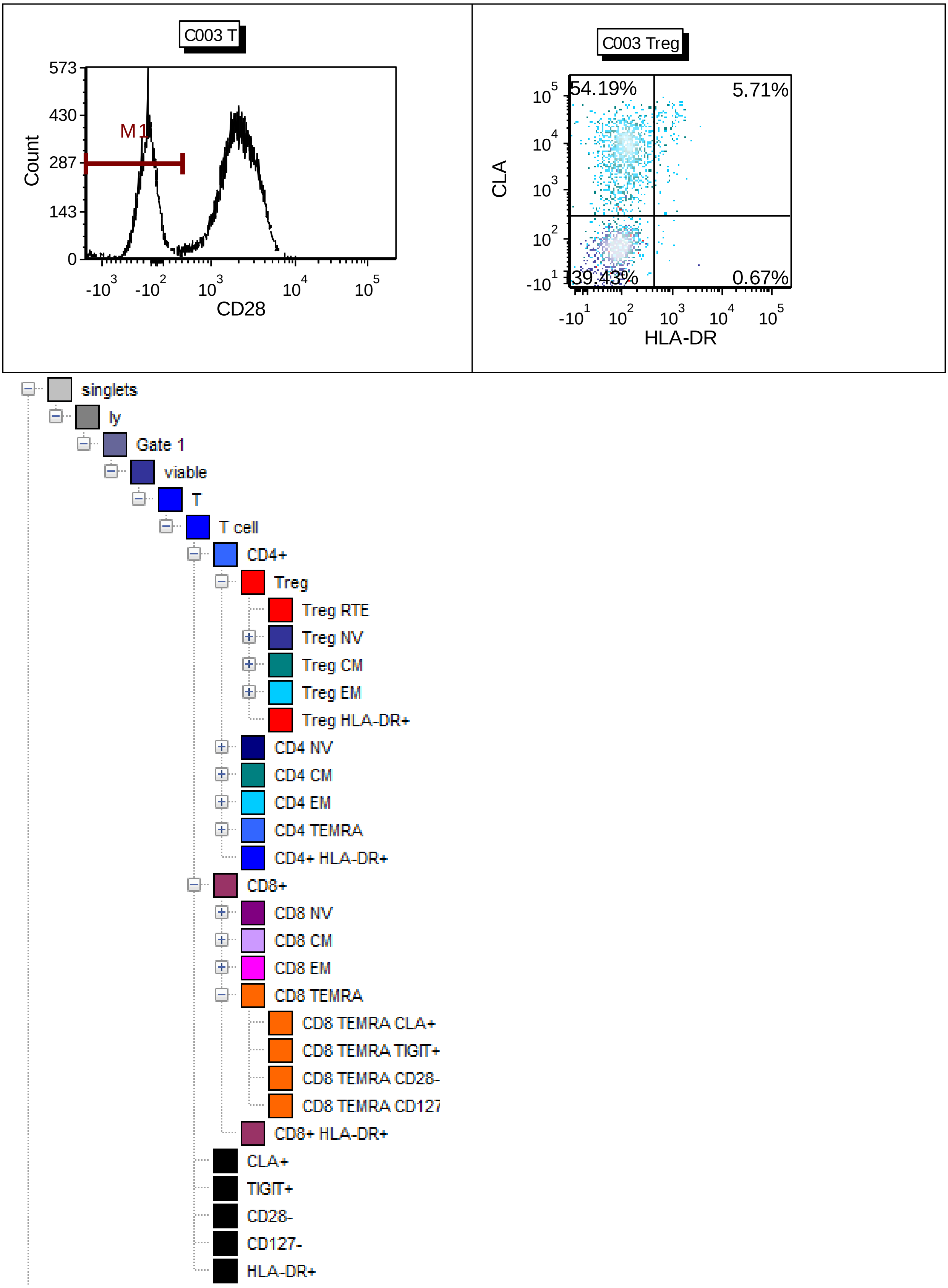

C003 T
573
430
287
143
0
Count
M1
-10^3
-10^2
10^3
10^4
10^5
CD28
C003 Treg
54.19%
5.71%
39.43%
0.67%
10^5
10^4
10^3
10^2
-10^1
CLA
-10^1
10^2
10^3
10^4
10^5
HLA-DR
singlets
ly
Gate 1
viable
T
T cell
CD4+
Treg
Treg RTE
Treg NV
Treg CM
Treg EM
Treg HLA-DR+
CD4 NV
CD4 CM
CD4 EM
CD4 TEMRA
CD4+ HLA-DR+
CD8+
CD8 NV
CD8 CM
CD8 EM
CD8 TEMRA
CD8 TEMRA CLA+
CD8 TEMRA TIGIT+
CD8 TEMRA CD28-
CD8 TEMRA CD127
CD8+ HLA-DR+
CLA+
TIGIT+
CD28-
CD127-
HLA-DR+
